# Effects of Short- And Medium-Term Exposures to Lower Air Temperature on 71 Novel Biomarkers of Subclinical Inflammation: Results from the KORA F4 Study

**DOI:** 10.1021/acs.est.3c00302

**Published:** 2023-08-08

**Authors:** Wenli Ni, Susanne Breitner, Nikolaos Nikolaou, Kathrin Wolf, Siqi Zhang, Annette Peters, Christian Herder, Alexandra Schneider

**Affiliations:** † Institute of Epidemiology, Helmholtz Zentrum München - German Research Center for Environmental Health (GmbH), Neuherberg D-85764, Germany; ‡ Institute for Medical Information Processing, Biometry, and Epidemiology, Pettenkofer School of Public Health, LMU Munich, Munich 81377, Germany; § Institute for Clinical Diabetology, German Diabetes Center, Leibniz Center for Diabetes Research at Heinrich Heine University Düsseldorf, Düsseldorf 40225, Germany; ∥ Division of Endocrinology and Diabetology, Medical Faculty and University Hospital Düsseldorf, Heinrich Heine University Düsseldorf, Düsseldorf 40204, Germany; ⊥ German Center for Diabetes Research (DZD), München-Neuherberg, Munich D-85764, Germany; # German Centre for Cardiovascular Research (DZHK), Partner Site Munich Heart Alliance, Munich 80802, Germany

**Keywords:** short- and medium-term effects, air temperature, inflammation, cytokines

## Abstract

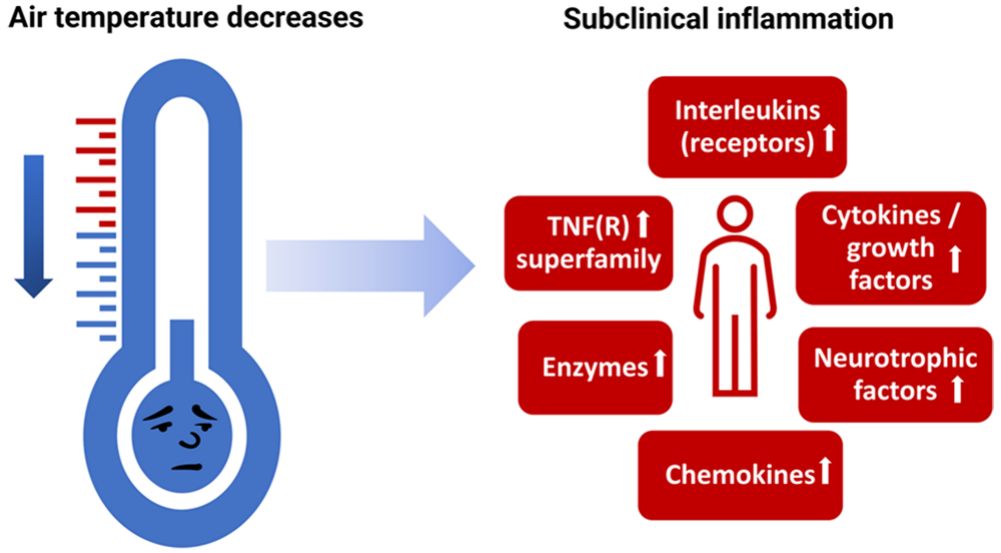

Increasing evidence has revealed that exposure to low temperatures is linked to a higher risk of chronic diseases and death; however, the mechanisms underlying the observed associations are still poorly understood. We performed a cross-sectional analysis with 1115 participants from the population-based KORA F4 study, which was conducted in Augsburg, Germany, from 2006 to 2008. Seventy-one inflammation-related protein biomarkers were analyzed in serum using proximity extension assay technology. We employed generalized additive models to explore short- and medium-term effects of air temperature on biomarkers of subclinical inflammation at cumulative lags of 0–1 days, 2–6 days, 0–13 days, 0–27 days, and 0–55 days. We found that short- and medium-term exposures to lower air temperature were associated with higher levels in 64 biomarkers of subclinical inflammation, such as Protein S100-A12 (EN-RAGE), Interleukin-6 (IL-6), Interleukin-10 (IL-10), C–C motif chemokine 28 (CCL28), and Neurotrophin-3 (NT-3). More pronounced associations between lower air temperature and higher biomarker of subclinical inflammation were observed among older participants, people with cardiovascular disease or prediabetes/diabetes, and people exposed to higher levels of air pollution (PM_2.5_, NO_2_, and O_3_). Our findings provide intriguing insight into how low air temperature may cause adverse health effects by activating inflammatory pathways.

## Abstract 2

The association between air temperature and biomarkers of inflammation has yet to be entirely understood. This study reports significant short- and medium-term effects of lower air temperature on higher biomarkers of subclinical inflammation.

## 1 Introduction

Climate change is an important public health issue that is characterized not only by an increasing frequency of extreme weather events but also by greater variability in temperature. Despite climate change, there will still be transient, unexpected temperature drops, and even if they are moderate and not extreme, they could still have an effect on health.^1^ Increasing evidence from epidemiological studies revealed a U-shaped association between the air temperature and mortality. Of note, mortality increases both above and below a certain temperature optimum that appears to vary geographically.^2−4^ Exposure to lower air temperature is also linked to a higher risk for chronic diseases.^5,6^ Low temperatures sometimes contribute to more deaths than high temperatures.^3,4,7−9^ For example, according to the Global Burden of Disease Study 2019, nonoptimal temperatures were a risk factor for global mortality, and low temperatures, compared to high temperatures, were associated with a greater mortality burden worldwide.^9^ A study based on more than 1 million clinical visits for inflammation-related diseases in the Haiyuan and Yanchi counties in China found that low air temperature exposure was associated with an increased risk of inflammation-related diseases.^10^

However, the mechanisms underlying the association between the air temperature and both chronic diseases and mortality still need to be better understood. Biomarkers of inflammation have been associated with the development of many chronic diseases.^11−13^ Several previous studies explored the association between air temperature and levels of biomarkers of inflammation.^14−21^ However these studies were limited in focusing on only a small number of biomarkers (e.g., Interleukin-6 [IL-6]), and the findings remained inconsistent. Furthermore, most previous studies either investigated only subgroups of the general population (e.g., myocardial infarction survivors) or were based on experimental or panel studies with a small number of participants (though the panel study had greater internal validity), thus limiting the possibility of controlling for confounding or of generalizing the results.

Inflammatory processes are complex and have been shown to play a role in various chronic diseases.^11,22^ Recent achievements in proteomic technologies have improved the detection of various inflammatory markers, but high-dimensional analyses between these markers and air temperature have not been conducted. To gain a more thorough understanding of how air temperature may cause adverse health effects through the systemic inflammatory pathway, we assessed short- and medium-term effects of air temperature (air temperature variability in space and time) on a multimarker panel of subclinical inflammation in a large population-based cohort in the Augsburg region, Germany. By analyzing a multimarker panel of biomarkers, we sought to capture a broad range of inflammatory effects and provide a more complete picture of the impact of the temperature on health. Furthermore, given that the different biomarkers of subclinical inflammation may have varying levels of sensitivity in detecting the effects of air temperature on health, we aimed to identify potential biomarkers that are more sensitive to temperature-related health responses, which may be recommended for use in future investigations.

## 2 Materials and Methods

### 2.1 Study Population

We performed a cross-sectional analysis using data from the Cooperative Health Research in the Region of Augsburg (KORA) F4 study (2006–2008), which was a follow-up study of the population-based KORA S4 survey conducted in the city of Augsburg (Southern Germany) and its two surrounding districts during 1999–2001.^23^ The latitude and longitude of Augsburg, Germany, are 48.366512 and 10.894446, respectively. The design of the study and data collection methods have been described in detail elsewhere.^23−26^ The current study is based on 1115 subjects aged 62 to 81 years for whom a multimarker panel of biomarkers of inflammation (see section 2.3) was available.

The study was approved by the Ethics Board of the Bavarian Chamber of Physicians (Munich, Germany) in adherence to the Declaration of Helsinki. All participants gave written informed consent.

### 2.2 Exposure Assessment

We estimated German-wide and highly resolved (1 km × 1 km) daily mean, minimum, and maximum air temperature using a multistage regression-based modeling approach.^27^ In order to achieve air temperature predictions with full spatial and temporal coverage across the country, we combined three stages. In the first stage, for days and grid cells where both monitor-based air temperature and satellite-derived land surface temperature were available, we trained a linear mixed effects model, including daily random intercepts and slopes for land surface temperature and several spatial predictors. In the second stage, we predicted air temperatures for grid cells without air temperature measurements but with available land surface temperature data using the model from the first stage. In the third stage, we regressed the second stage air temperature predictions against thin plate spline interpolated air temperature values for all remaining days and grid cells with neither air temperature measurements nor satellite land surface temperature available. In order to evaluate the performance of our models, we applied internal and external 10-fold cross-validation. All models showed excellent performance (0.91 ≤ *R*^2^ ≤ 0.98) and low errors (1 °C < Root Mean Square Error (RMSE) < 2 °C). Especially in Augsburg, Germany, we extensively evaluated our model predictions by comparing them with measurements from an independent network of 82 HOBO-Logger devices^28^ with similarly good results (0.94 ≤ *R*^2^ ≤ 0.99, 0.99 °C ≤ RMSE ≤ 1.87 °C).

Details of the assessment of relative humidity (RH), particulate matter with an aerodynamic diameter <2.5 μm (PM_2.5_), nitrogen dioxide (NO_2_), and ozone (O_3_) are given in Text S1 in the Supporting Information.

### 2.3 Measurement of Novel Systemic Biomarkers of Subclinical Inflammation

The OLINK Inflammation multiplex immunoassay (OLINK Proteomics, Uppsala, Sweden) used in this study includes 92 inflammation-related protein biomarkers from serum samples: pro- and anti-inflammatory cytokines, chemokines, growth factors, and factors involved in acute inflammatory and immune responses, angiogenesis, fibrosis, and endothelial activation. Serum samples were stored at −80 °C and thawed once before shipment to the OLINK Proteomics for biomarker measurement, which was performed in 2016–2017. The measurements were based on the proximity extension assay (PEA) technology that binds oligonucleotide-labeled antibody probe pairs to the respective target protein in the sample and uses a polymerase chain reaction for signal amplification.^29^ The relative quantification of protein levels was expressed as normalized protein expression (NPX) arbitrary units on a Log2 scale. Details of the assessment of covariates are given in Text S2 in the Supporting Information.

An overview of all 92 analytes, including assay ID, abbreviated, full names, UniProt numbers, intra-assay coefficient of variation (CV), interassay CV, and limit of detection (LOD), is given in Table S1 (Supporting Information). As described before,^30^ the intra- and interassay CVs were calculated based on the three control sera measured in duplicates on each plate (*n* = 16). Twenty biomarkers were excluded because of ≥25% of samples below the limit of detection (LOD), and one additional biomarker was excluded because of an interassay CV > 20%. For the remaining 71 analytes, sample values below the LOD were set to the LOD. In the final data set (71 biomarkers), the intra-assay CV was 3.6 ± 1.5% (mean ± SD), and the interassay CV was 8.4 ± 2.2% (mean ± SD).

### 2.5 Statistical Analysis

The characteristics of the study population were reported by frequency and percentage for categorical variables and mean and standard deviation (SD) for continuous variables. The levels of biomarkers of subclinical inflammation, meteorological variables, and air pollutants were summarized as mean, SD, 5%, 25%, median, 75%, and 95% percentiles. Spearman correlation analysis was used to evaluate the correlations.

We employed generalized additive models (GAMs) to explore short- and medium-term effects of the mean air temperature on biomarkers of subclinical inflammation. Biomarkers values outside of three times the interquartile range were excluded to avoid bias due to the presence of outliers. In order to explore the lagged and cumulative effects of air temperature, we investigated the effects of mean air temperature at 0–1, 2–6, and 0–13 days before blood draw for short-term effects and 0–27, and 0–55 days before blood draw for medium-term effects. Almost no appreciable deviations from linearity were found for exposure-response functions (spline with three degrees of freedom), so air temperature was included linearly in the GAMs (Figure S1, Supporting Information).

We controlled for potential confounders based on published literature and expert knowledge:^11,31^ age, sex, education, smoking status, alcohol consumption, physical activity, height, waist circumference, systolic blood pressure, diastolic blood pressure, albumin, hematocrit, day of the week, season at blood draw (cold: April–September, warm: October–March), time trend (cubic spline with six degrees of freedom per year), and RH (cubic spline with three degrees of freedom) with the same lag period as the air temperature.

The results were expressed as percent changes of the outcome mean (with their 95% confidence intervals [CIs]) per 1-interquartile range (IQR) decrease in air temperature. We adjusted for multiple testing of different exposure windows and biomarkers of subclinical inflammation using the Benjamin-Hochberg false discovery rate (FDR). *P* (adjusted)-value <0.05 was considered statistically significant for all statistical tests.

For biomarkers of subclinical inflammation showing significant (adjusted *p*-value <0.05) associations with air temperature, further stratification analyses were conducted to examine effect modification by age (<70 years vs ≥70 years), sex (male vs female), cardiovascular disease (defined as a history of hypertension, angina pectoris, stroke, or myocardial infarction [yes vs no]), (pre)diabetes status (normal glucose tolerance vs prediabetes/diabetes), air pollutants with the same lag period as the air temperature (PM_2.5_/NO_2_/O_3_: low [<median] vs high [ ≥ median]).

We performed several sensitivity analyses to assess the robustness of our results further. First, we additionally adjusted for medication intake (antihypertensive drugs or nonsteroidal anti-inflammatory drugs) in the main model. Second, we controlled for the presence of pre-existing conditions (cardiovascular disease, cancer, and chronic bronchitis or emphysema) by incorporating them as additional adjustment factors. Third, to control for potential confounding by air pollutants, the concentrations of three pollutants (PM_2.5,_ NO_2_, and O_3_ [continuous variables]) were additionally included in the main model, though separately, to avoid collinearity. Fourth, we accounted for wind speed and barometric pressure in the main model as additional adjustment factors. Fifth, participants with C-reactive protein (CRP) values greater than 10 mg/L were excluded (*N* = 47) because this might indicate acute infection. Sixth, we used the minimum and maximum air temperatures instead of the mean air temperature. Finally, to control for the confounding effect of season, we linearly regressed season on the biomarkers of subclinical inflammation and then calculated the respective residuals for further association analyses with air temperature.

All statistical analyses were performed by using R (version 4.1.2) with the “mgcv” package.

## 3 Results

### 3.1 Study Population, Biomarkers of Inflammation, and Exposure Data

Table 1 describes the characteristics of the study population. The mean age in this study population was 70.4 years, 48.8% of the study population was female, 39.8% reported low physical activity, and only 7.4% were current smokers.

**Table 1:** Descriptive Statistics of Participant Characteristics^a^

	Mean ± SD/N (%) (*n* = 1115)
**Age (years)**	70.4 ± 5.5
**Sex (female)**	544 (48.8%)
**Education (years)**	11.0 ± 2.5
**Smoking status**	
Current smoker	82 (7.4%)
Former smoker	486 (43.6%)
Nonsmoker	544 (48.8%)
**Physical activity**	
Low	444 (39.8%)
Medium	429 (38.5%)
High	239 (21.4%)
**Height (cm)**	166 ± 9.0
**Waist circumference (cm)**	98.3 ± 12.2
**Body mass index**(kg/m^2^)	28.7 ± 4.5
**Systolic blood pressure (mmHg)**	129 ± 19.8
**Diastolic blood pressure (mmHg)**	74.1 ± 10.0
**Albumin**(g/L)	43.7 ± 3.2
**Haematocrit**(L/L)	0.4 ± 0.03
**Alcohol consumption (g/day)**	13.8 ± 18.1
**Cardiovascular disease (yes)**	741 (66.5%)
**Cancer (yes)**	156 (14.0%)
**Chronic bronchitis or emphysema (yes)**	131 (11.7%)
**Diabetes status**	
Normal glucose tolerance	577 (51.7%)
Prediabetes	284 (25.5%)
Diabetes	231 (20.7%)
**Medication intake**	
Antihypertensive medication (yes)	657 (58.9%)
NSAIDs (yes)	48 (4.3%)
**Season of examination**	
Cold	744 (66.7%)
Warm	371 (33.3%)

a Note: SD: Standard deviation. Physical activity: low, almost no activity; medium, regularly or irregularly about 1 h per week; high, regularly more than 2 h per week. NSAIDs: Nonsteroidal anti-inflammatory drugs. Season: cold, April–September; warm, October–March.

Levels of biomarkers of subclinical inflammation are presented in Figure S2 and Table S2 (Supporting Information). Almost all correlations among the biomarkers of subclinical inflammation were positive and, in most cases, with low to moderate Spearman coefficients (Figure S3, Supporting Information).

Table 2 summarizes the meteorological variables and air pollutant levels to which our participants were exposed during the study period. The mean level of mean air temperature was 7.8 ± 6.1 °C (mean ± SD). Figure S4 shows the time series of mean air temperatures for participants in this study. High correlations were observed between air temperature variables (mean, minimum, and maximum air temperature) as well as between PM_2.5_, NO_2,_ and wind speed, while weak to moderate correlations were observed between other meteorological variables and other air pollutants (Figure S5, Supporting Information).

**Table 2:** Descriptive Statistics of Meteorological Variables and Air Pollutants^b^

	Mean ± SD	Min	25%	Median	75%	Max
Mean air temperature (°C)	7.8 ± 6.1	–7.8	2.8	7.1	11.8	24.7
Minimum air temperature (°C)	3.8 ± 5.4	–13.2	0.0	3.0	7.0	17.1
Maximum air temperature (°C)	12.4 ± 7.5	–4.6	7.1	11.5	17.4	35.0
RH (%)	77.0 ± 9.9	46.5	70.9	78.2	84.0	94.5
PM_2.5_ (μg/m^3^)	14.8 ± 11.2	1.4	6.1	12.6	19.7	65.8
O_3_ (μg/m^3^)	38.6 ± 22.8	3.0	18.7	36.0	54.8	97.6
NO_2_ (μg/m^3^)	33.3 ± 11.9	10.4	23.3	32.4	41.2	77.9
Wind speed (m/s)	3.5 ± 2.1	0.9	1.9	3.0	4.4	15.3
Barometric pressure (hPa)	1018.1 ± 8.2	996.5	1013.0	1018.4	1023.9	1037.6

b Note: RH, relative humidity; PM_2.5_, particulate matter with an aerodynamic diameter of ≤2.5 μm; O_3_, ozone; NO_2_, nitrogen dioxide.

### 3.2 Short- and Medium-Term Effects of Air Temperature on 71 biomarkers of Subclinical Inflammation

**Figure 1:**
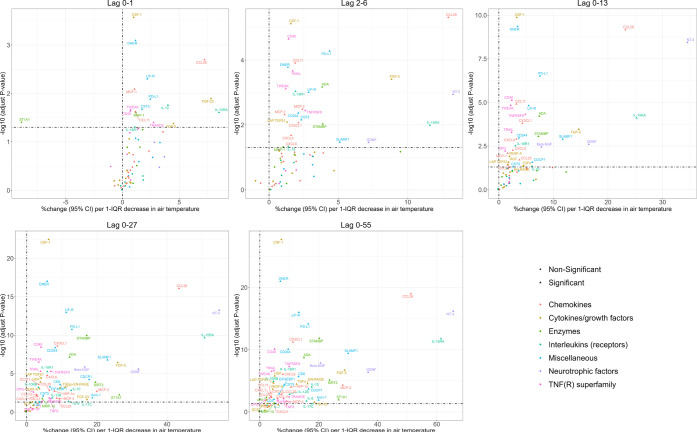
Volcano plots presenting the associations between short- and medium-term exposures to air temperature per 1-IQR decrease with 71 biomarkers of subclinical inflammation. Note: the 1-IQR decrease was 9.2 °C for lags 0–1 days, 8.9 °C for lags 2–6 days, 8.4 °C for lags 0–13 days, 9.0 °C for lags 0–27 days, and 9.4 °C for lags 0–55 days.

**Figure 2:**
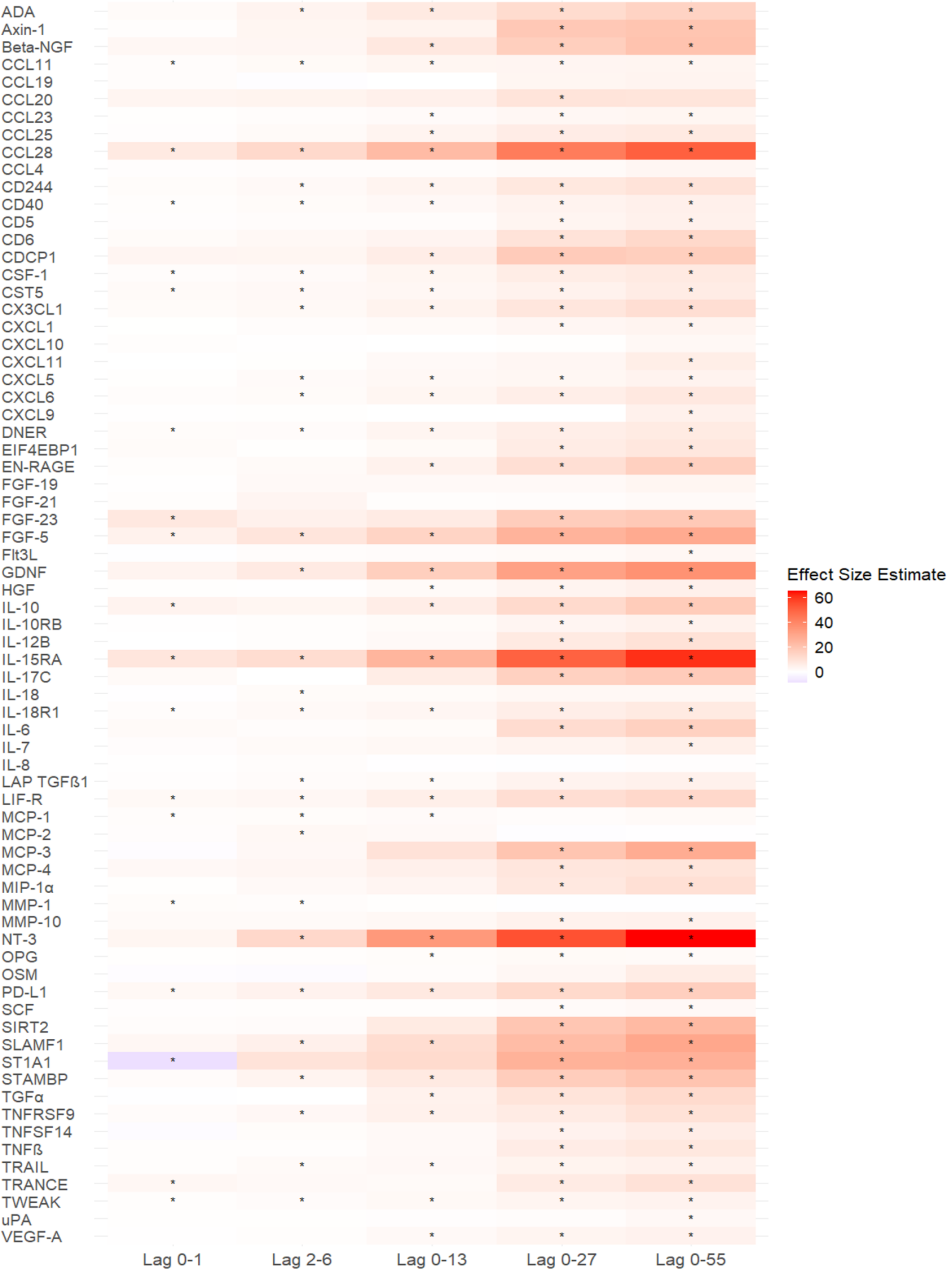
Heatmap of associations between short- and medium-term exposure to air temperature and 71 biomarkers of subclinical inflammation. Note: * *P* (adjusted)-value <0.05, Effect size estimate: percent changes of the outcome mean per 1-IQR decrease in air temperature.

The short- and medium-term effects of air temperature on 71 biomarkers of subclinical inflammation are shown in Figure 1 and 2, Figures S6 and S7 (Supporting Information). For a brief overview, a 1-IQR decrease in air temperature was significantly associated with increases in 64 biomarkers of subclinical inflammation (40 significant associations for short-term effects and 60 significant associations for medium-term effects), such as Protein S100-A12 (EN-RAGE), IL-6, Interleukin-10 (IL-10), C–C motif chemokine 28 (CCL28), Neurotrophin-3 (NT-3), and Interleukin-15 receptor subunit alpha (IL-15RA). Of these significant associations, there were associations for 17 biomarkers of subclinical inflammation at lag 0–1 days, 28 biomarkers of subclinical inflammation at lag 2–6 days, 35 biomarkers of subclinical inflammation at lag 0–13 days, 55 biomarkers of subclinical inflammation at lag 0–27 days, and 59 biomarkers of subclinical inflammation at lag 0–55 days. The number of significant associations with biomarkers of subclinical inflammation and their effect estimates increased with an increasing number of lag days. Figure S8 summarizes the associations between short- and medium-term exposures to air temperature per 1 °C decrease with 71 biomarkers of subclinical inflammation.

Venn diagrams (Figure S9, Supporting Information) show 13 overlapping biomarker associations at different exposure windows for short-term effects, 54 overlapping biomarker associations at different exposure windows for medium-term effects, and 12 overlapping biomarker associations (Eotaxin [CCL11], CCL28, CD40L receptor [CD40], Macrophage colony-stimulating factor 1 [CSF-1], Cystatin D [CST5], Delta and Notch-like epidermal growth factor-related receptor [DNER], Fibroblast growth factor 5 [FGF-5], IL-15RA, Interleukin-18 receptor 1 [IL-18R1], Leukemia inhibitory factor receptor [LIF-R], Programmed cell death 1 ligand 1 [PD-L1], and Tumor necrosis factor [Ligand] superfamily, member 12 [TWEAK]) at different exposure windows for short- and medium-term effects. The significant biomarker associations found at lag 0–27 days were almost completely replicated at lag 0–55 days.

#### 3.3 **Effect Modification**

**Figure 3:**
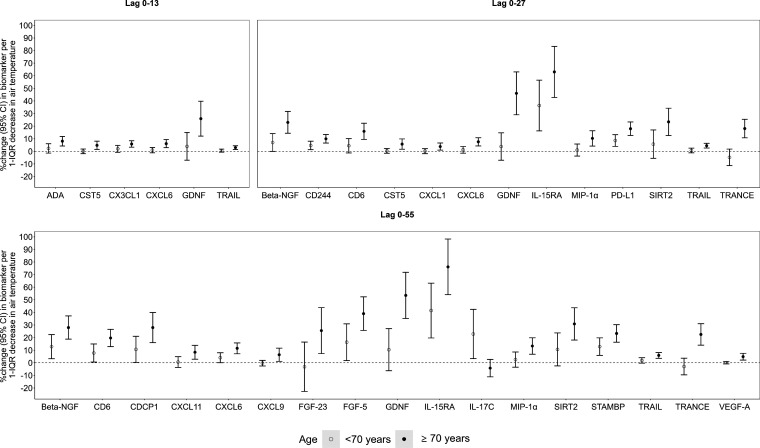
Short- and medium-term effects of air temperature on biomarkers of subclinical inflammation per 1-IQR decrease significantly modified by age. Note: the 1-IQR decrease was 8.4 °C for lags of 0–13 days, 9.0 °C for lags of 0–27 days, and 9.4 °C for lags of 0–55 days.

**Figure 4:**
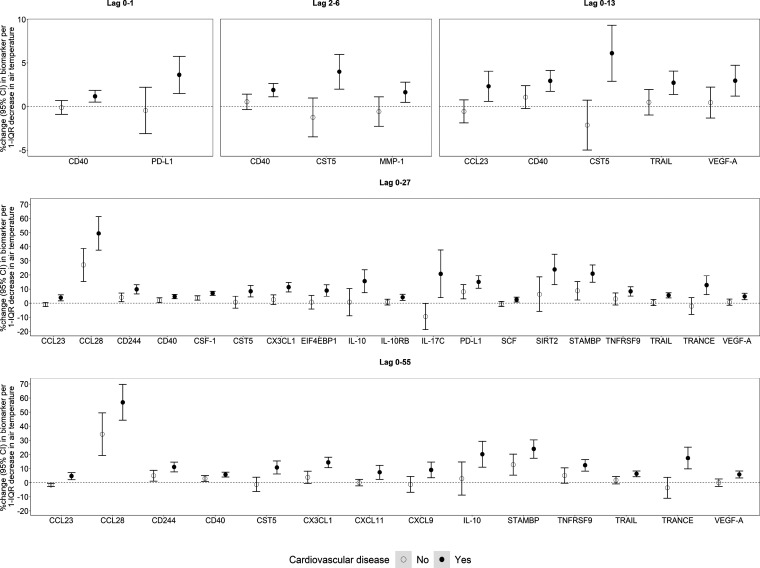
Short- and medium-term effects of air temperature on biomarkers of subclinical inflammation per 1-IQR decrease significantly when modified by cardiovascular disease. Note: the 1-IQR decrease was 9.2 °C for lags 0–1 days, 8.9 °C for lags 2–6 days, 8.4 °C for lags 0–13 days, 9.0 °C for lags 0–27 days, and 9.4 °C for lags 0–55 days.

We found stronger effects of air temperature (i) on 22 biomarkers (e.g., Beta-nerve growth factor [Beta-NGF], C-X-C motif chemokine 6 [CXCL6], Glial cell line-derived neurotrophic factor [GDNF], IL-15RA, TNF-related apoptosis-inducing ligand [TRAIL], and TNF-related activation-induced cytokine [TRANCE]) in participants older than 70 years of age, (ii) on 22 biomarkers (e.g., C–C motif chemokine 23 [CCL23], CCL28, CST5, IL-10, TRAIL, and TRANCE) in participants with cardiovascular disease, and (iii) on 11 biomarkers (e.g., CCL28, CST5, and Interleukin-10 receptor subunit beta [IL-10RB]) in participants with prediabetes/diabetes compared to their respective counterparts (Figure 3, Figure 4, and Figure S10 [Supporting Information]). We also found stronger effects of air temperature on eight biomarkers (e.g., FGF-5, Neurotrophin-3 [NT-3]) in men than in women and on five biomarkers (e.g., IL-6) in women than in men (Figure S10, Supporting Information). Finally, we found stronger effects of air temperature on 21, 44, and 20 biomarkers in participants’ exposure to higher levels of PM_2.5_, NO_2_, and O_3_, respectively, than in those exposed to lower levels (Figures S11 and S12, Supporting Information).

#### 3.4 **Sensitivity Analysis**

Overall, the results of the sensitivity analyses were consistent with those of the main analysis (data not shown). First, similar effect estimates were seen when additionally adjusting for medications, pre-existing conditions, air pollutants, or wind speed and barometric pressure in the model. Moreover, excluding study participants with CRP values greater than 10 mg/L did not affect the results (Figure S13, Supporting Information). Third, using minimum or maximum temperatures instead of the mean provided similar results. Finally, the findings were consistent when we used the residuals of the linear regression of season on biomarkers of subclinical inflammation.

### 4 Discussion

#### 4.1 **Summary of Key Results**

To the best of our knowledge, this is the first study to investigate the effects of short- and medium-term exposures to air temperature on a multimarker panel of biomarkers of subclinical inflammation. Among the 71 biomarkers of subclinical inflammation, a lower air temperature showed statistically significant associations with higher levels in 64 biomarkers, after controlling for extensive potential confounding factors and correction for multiple tests.

#### 4.2 **Comparison with Current Evidence**

Of the 71 biomarkers of subclinical inflammation reported in this study, only 5 of them (IL-6, Interleukin-8 [IL-8], IL-10, Monocyte chemotactic protein 1 [MCP-1], and Fibroblast growth factor 21 [FGF-21]) have previously been reported in association with air temperature in epidemiology studies, and most of these studies only explored short-term effects.^14−21^ We found that a 1-IQR decrease in air temperature was significantly associated with higher levels of IL-6, IL-10, and MCP-1 for different lag windows up to 0–55 days. In contrast, there were no significant associations between the air temperature and IL-8 or FGF-21. Previous studies on older people (aged 60–82 years) or myocardial infarction survivors found that decreased air temperature was associated with increased IL-6 level at lag 1 day or 5-day moving average.^17,18^ A panel study with a specific genetic background from KORA F4 study showed also decreased air temperature was associated with increased IL-6 level at lag 1 day, lag 4 day, and 5-day moving average; at the same time, no significant associations were seen in 187 people with type 2 diabetes and impaired glucose tolerance.^14^ In addition, lower air temperature was associated with higher IL-6, IL-8, IL-10, or MCP-1 at lag 0 to lag 2 days in 35 people with type 2 diabetes in Shanghai, China.^16^ Another study based on 77 healthy volunteers in North Carolina, United States, found that IL-8, but not MCP-1, was inversely associated with the air temperature of the previous day.^19^ A crossover intervention study of 12 volunteers found that FGF-21 was higher at 19 °C than at 24 °C after 3–9 h.^20^ In contrast, another experimental study of 19 healthy participants found that FGF-21 significantly decreased after 2 h of cold exposure in brown adipose tissue (BAT)-positive subjects, but not BAT-negative subjects.^21^ Moreover, a study only focusing on men in the Greater Boston area, United States, found no associations between air temperature and IL-6 or IL-8 at lags 0 to 7, and 1-, 2-, 3-, and 4-week moving averages.^15^ Therefore, our results of inverse associations between the air temperature and the biomarkers in our population-based setting mostly align with the current evidence from smaller studies based on selected populations and less extensive analyses of different lag times.

#### 4.3 **Duration of Effects**

We found that medium-term effects of air temperature were stronger than short-term effects, with more significant biomarker associations and larger effect estimates. These findings suggest delayed effects of the lower air temperature on these biomarkers of subclinical inflammation. The larger effect sizes of the medium-term exposures could also be due, in part, to the cumulative impact of the low-temperature exposures. Of note, most previous studies on air temperature and inflammation have only investigated short-term associations, and the results of our study suggest these may be underestimated. Given that many of the temperature-sensitive biomarkers of inflammation have been related to the risk of various diseases and mortality (see section 4.6), our study indicates that adverse health effects of lower air temperature may not only be acute but relevant over at least two months.

#### 4.4 **Highlighted Biomarkers**

Of the biomarkers we analyzed, (i) NT-3, IL-15RA, CCL28, FGF-5, and GDNF were about the top five biomarkers with the largest effects; (ii) CCL11, CCL28, CD40L CD40, CSF-1, CST5, DNER, FGF-5, IL-15RA, IL-18R1, LIF-R, PD-L1, and TWEAK were significantly associated with short- and medium-term effects across all exposure windows. These findings indicate that these potential biomarkers may be more sensitive to temperature-related health responses and may be better recommended in future studies for the detection of temperature-related adverse health responses.

#### 4.5 **Novel Associations**

To the best of our knowledge, no epidemiological study investigated the effects of air temperature on the other 66 biomarkers of subclinical inflammation reported in our study. Hence, we substantially extended the current literature in this field. The present study identified significant associations for 64 biomarkers of subclinical inflammation, 61 of which were reported for the first time to be associated with lower air temperature exposures. In our analyses, we adjusted for a range of potential confounders and also for multiple testing (different exposure windows and biomarkers of subclinical inflammation). Covariates included not only standard demographic, anthropometric, and metabolic variables but also albumin and hematocrit to adjust for potential confounding effects between air temperature and changes in blood volume due to vasoconstriction and vasodilation. Furthermore, consistent results were obtained from multiple sensitivity analyses. We especially excluded participants with markedly high CRP values (>10 mg/L) who might have had an acute infection. The results remained stable, suggesting that these significant associations were not due to the confounding effects of acute infection. We used two exposure windows for medium-term effects and found nearly identical effects, again illustrating the stability of our results.

Our study may have several clinical implications. These are related to the (i) lag times, and thus the duration of temperature effects on subclinical inflammation, (ii) biomarkers that are regulated and have been previously found to be associated with morbidity and mortality, (iii) identification of subgroups within the population that show more pronounced responses to temperature changes than others, and (iiii) identification of interactive effects between lower air temperature and higher air pollution exposures on increased biomarkers of subclinical inflammation.

#### 4.6 **Mechanisms Linking Lower Air Temperature to Morbidity and Mortality**

Exposure to low temperatures is associated with not only increased risks of various chronic diseases but also increased mortality.^6,8,32,33^ Many previous studies reported that increased pro- and anti-inflammatory biomarkers (IL-6, EN-RAGE, and IL-10) were associated with increased risks of diabetes, cardiovascular disease, and mortality.^34−42^ IL-6 is a pleiotropic cytokine with pro-inflammatory effects, which can induce atherosclerosis in cardiovascular disease.^43^ EN-RAGE binds to RAGE, activating the pro-inflammatory NF-κB signaling, the typical innate immune system pathway involved in coronary heart disease pathogenesis.^36,44^ IL-10 is a pleiotropic cytokine that is most widely recognized as an anti-inflammatory cytokine. However, previous studies found that upregulation of IL-10 was positively associated with the risk of cardiovascular events, although the association was not consistent.^38−40^ Many of the other novel biomarkers of subclinical inflammation in this study are exploratory. However, they point toward cell–cell communication (e.g., chemokines involved in the cross-talk between innate and adaptive immunity), a role in immune responses, and neurological processes. Of note, the same assay allowed the identification of multiple biomarkers of inflammation associated with incident distal sensorimotor polyneuropathy^30^ and impaired kidney function,^45,46^ many of which were temperature-responsive in this study. In summary, our findings raise the possibility that lower air temperature exposure could affect the risk of multiple age-related and chronic diseases in addition to mortality partly through the effect on subclinical inflammation.

#### 4.7 **Susceptible Subgroups**

We found that the effects of air temperature on biomarkers of subclinical inflammation were stronger in participants ≥70 years compared to participants <70 years. This may be related to the decline of body function and the thermoregulatory capacity in the elderly with age.^47,48^ Also, people with underlying health conditions, such as cardiovascular disease and diabetes, were more vulnerable to temperature decreases. These observations are consistent with previous reports that low air temperature exposures increase the risks for both conditions.^6,49,50^ Our findings lead to the hypothesis that subclinical inflammation may be one of the underlying mechanisms behind the associations of low temperature with cardiometabolic disease, which merits further studies in the future. Interestingly, we did not find evidence for consistent effect modification by sex (stronger effects of the air temperature on eight biomarkers in men and five biomarkers in women). Several previous studies found that the mortality among men exposed to low temperatures was higher than that among women,^51−53^ while other studies showed opposite findings.^48,54^ Overall, the role of sex in modifying temperature–mortality associations is not clear, which could at least partly be due to differential effects of air temperature on different biomarkers of subclinical inflammation that vary according to sex.

#### 4.8 **Interactive Effects between Lower Air Temperature and Higher Air Pollution**

Strikingly, we found interactive effects between a lower air temperature and higher air pollution exposures on increased biomarkers of subclinical inflammation. Our findings suggest that given the adverse health effects of low temperature, the synergy of low temperature and air pollution aggravates the impact on health. Previous studies have showed that the potential interactive effect of low temperature and air pollution exposure on cardiovascular diseases have been found.^55−57^ More importantly, lower air temperatures and higher air pollution exposures often coexist due to extensive use of coal, wood, diesel, or oil burn for heating during the colder temperature in many parts of the world, e.g., China. These highlights that integrated climate and air quality policies should be formulated to strengthen the response to the combined health threats of temperature and air pollution.

#### 4.9 **Limitations**

Our study also has limitations. First, we conducted this study in one study area, limiting its generalizability beyond the study area but, most importantly, to areas in colder or warmer climate zones. Further cohort studies with similar research designs are necessary to corroborate our findings. Second, we used area-level exposure estimates rather than individual measurements, which may have led to a possible bias due to the misclassification of exposure and a potential underestimation of true associations. Third, as a cross-sectional study, it lacks the ability to observe changes over time. Additionally, despite adjusting for a range of covariates, residual confounding or unmeasured confounders may still be present. Finally, protein degradation is possible with long-term storage, but is expected to be nondifferential, i.e., should not depend on the air temperature exposure, which was the main exposure of the analyses. Therefore, the effects on our results are extremely unlikely.

In conclusion, we found that short- and medium-term exposures to lower air temperature were associated with higher levels in 64 biomarkers of subclinical inflammation, such as EN-RAGE, IL-6, IL-10, CCL28, and NT-3, some of which have been related to a higher risk of chronic diseases or mortality before. Our findings provide more insight into the complexity of the relationship between low air temperatures and adverse health effects and indicate that subclinical inflammation could be a relevant mediator meriting further studies

## Supporting Information Available

Text S1, assessment of meteorological variables and air pollutants; Text S2, assessment of covariates; Table S1, biomarkers of subclinical inflammation in the OLINK Inflammation panel and assay characteristics; Table S2, levels of 71 biomarkers of subclinical inflammation in serum; Figure S1, exposure-response functions of air temperature and biomarkers of subclinical inflammation at lags 0–13 days; Figure S2, the levels of 71 biomarkers of subclinical inflammation in serum samples; Figure S3, spearman correlation between biomarkers of subclinical inflammation; Figure S4, time series of daily mean air temperature for participants in this study; Figure S5, correlation between meteorological variables and air pollutants; Figure S6, significant associations between short-term exposure to air temperature per 1-IQR decrease with biomarkers of subclinical inflammation (*P*-adjust <0.05); Figure S7, significant associations between medium-term exposure to air temperature per 1-IQR decrease with biomarkers of subclinical inflammation (*P*-adjust <0.05); Figure S8, volcano plots presenting the associations between short- and medium-term exposures to air temperature per 1 °C decrease with 71 biomarkers of subclinical inflammation; Figure S9, Venn diagrams of significant associations between short- and medium-term exposures to air temperature per 1-IQR decrease with biomarkers of subclinical inflammation; Figure S10, short- and medium-term effects of air temperature on biomarkers of subclinical inflammation per 1-IQR decrease significantly modified by sex or diabetes; Figure S11, short- and medium-term effects of air temperature on biomarkers of subclinical inflammation per 1-IQR decrease significantly modified by PM_2.5_ or O_3_; Figure S12, short- and medium-term effects of air temperature on biomarkers of subclinical inflammation per 1-IQR decrease significantly modified by NO_2_; Figure S13, sensitivity analysis (participants with CRP values >10 mg/L were excluded): significant associations between short- and medium-term exposures to air temperature per 1-IQR decrease with biomarkers of subclinical inflammation (PDF)
