# Supplementary material for: Effects of Short- And Medium-Term Exposures to Lower Air Temperature on 71 Novel Biomarkers of Subclinical Inflammation: Results from the KORA F4 Study
Source: Environ Sci Technol. 2023 Aug 8;57(33):12210–21. doi: 10.1021/acs.est.3c00302 (PMC10448716; doi:10.1021/acs.est.3c00302)
Supplement: Supplementary file 1 — es3c00302_si_001.pdf [file es3c00302_si_001.pdf]

At Supporting Information for

**Effects of short- and medium-term exposures to lower air temperature on 71 novel biomarkers of subclinical inflammation: results from the KORA F4 study**

**Authors:** Wenli Ni[1,2], Susanne Breitner[1,2], Nikolaos Nikolaou[1,2], Kathrin Wolf[1], Siqi Zhang[1], Annette Peters[1,2,5,6], Christian Herder[3,4,5$], Alexandra Schneider[1$]

[1] Institute of Epidemiology, Helmholtz Zentrum München - German Research Center for Environmental Health (GmbH), Neuherberg, D-85764, Germany.

[2] Institute for Medical Information Processing, Biometry, and Epidemiology, Pettenkofer School of Public Health, LMU Munich, Munich, 81377, Germany

[3] Institute for Clinical Diabetology, German Diabetes Center, Leibniz Center for Diabetes Research at Heinrich Heine University Düsseldorf, Düsseldorf, 40225, Germany.

[4] Division of Endocrinology and Diabetology, Medical Faculty and University Hospital Düsseldorf, Heinrich Heine University Düsseldorf, Düsseldorf, 40204, Germany.

[5] German Center for Diabetes Research (DZD), München-Neuherberg, Munich, D-85764, Germany.

[6] German Centre for Cardiovascular Research (DZHK), Partner Site Munich Heart Alliance, Munich, 80802, Germany.

[$] C.H. and A.S. made equal contributions and share last authorship.

**Corresponding author:**

Wenli Ni

Institute of Epidemiology, Helmholtz Zentrum München - German Research Center for Environmental Health (GmbH), Ingolstädter Landstr. 1, D-85764 Neuherberg, Germany.

Institute for Medical Information Processing, Biometry, and Epidemiology, Pettenkofer School of Public Health, LMU Munich, Munich, Germany.

E-Mail: wenli.ni@helmholtz-muenchen.de

Pages: 38

Tables: 2

Figures: 13

## Table of Contents

Text S1. Assessment of meteorological variables and air pollutants

Text S2. Assessment of covariates

Table S1. Biomarkers of subclinical inflammation in the OLINK Inflammation panel and assay characteristics.

Table S2. Levels of 71 biomarkers of subclinical inflammation in serum.

Figure S1. Exposure-response functions of air temperature and biomarkers of subclinical inflammation at lags 0-13 days.

Figure S2. The levels of 71 biomarkers of subclinical inflammation in serum samples.

Figure S3. Spearman correlation between biomarkers of subclinical inflammation.

Figure S4. Time series of daily mean air temperature for participants in this study.

Figure S5. Correlation between meteorological variables and air pollutants.

Figure S6. Significant associations between short-term exposure to air temperature per 1-IQR decrease with biomarkers of subclinical inflammation (P-adjust <0.05).

Figure S7. Significant associations between medium-term exposure to air temperature per 1-IQR decrease with biomarkers of subclinical inflammation (P-adjust <0.05).

Figure S8. Volcano Plots presenting the associations between short- and medium-term exposures to air temperature per 1°C decrease with 71 biomarkers of subclinical inflammation.

Figure S9. Venn diagrams of significant associations between short- and medium-term exposures to air temperature per 1-IQR decrease with biomarkers of subclinical inflammation.

Figure S10. Short- and medium-term effects of air temperature on biomarkers of subclinical inflammation per 1-IQR decrease significantly modified by sex or diabetes.

Figure S11. Short- and medium-term effects of air temperature on biomarkers of subclinical inflammation per 1-IQR decrease significantly modified by $PM_{2.5}$ or $O_3$.

Figure S12. Short- and medium-term effects of air temperature on biomarkers of subclinical inflammation per 1-IQR decrease significantly modified by $NO_2$.

Figure S13. Sensitivity analysis (participants with CRP values > 10 mg/L were excluded): Significant associations between short- and medium-term exposures to air temperature per 1-IQR decrease with biomarkers of subclinical inflammation.

**Text S1. Assessment of meteorological variables and air pollutants**

Relative humidity (RH), particulate matter with an aerodynamic diameter <2.5 μm ($PM_{2.5}$), nitrogen dioxide ($NO_2$), ozone ($O_3$), wind speed, and barometric pressure were measured at fixed urban background monitoring sites operated by the Bavarian Environment Agency (LfU, Bayerisches Landesamt für Umwelt) in Augsburg, Germany.[1,2] Using Tapered Element Oscillating MicroBalance (TEOM model 1400A, ThermoFisherScientific) equipped with the Filter Dynamics Measurement System (FDMS, model 8500b; ThermoFisherScientific), $PM_{2.5}$ was measured at a single urban background site located 1 km south of the city center. The monitoring site for $NO_2$ was located about 2km north of the city center, and the monitoring sites for RH, $O_3$, wind speed, and barometric pressure were located about 5 km south of the city center. At least 75% of the hourly measurements had to be available to calculate the daily 24-hour average $NO_2$, maximum 8-hour average $O_3$, 24-hour average $PM_{2.5}$, and 24-hour average meteorological variables, respectively.

**Text S2. Assessment of covariates**

Information on participants' sociodemographic characteristics (age and sex), lifestyle (smoking status, alcohol intake, and physical activity), history of chronic diseases (hypertension, angina pectoris, stroke, myocardial infarction, and diabetes) and current use of medication (antihypertensive and nonsteroidal anti-inflammatory drugs), was collected via a computer-assisted personal interview and a self-administered questionnaire. Anthropometric measurements (height, body weight, and waist circumference) and blood pressure (systolic blood pressure, diastolic blood pressure) were measured during the physical examination.

Smoking was dichotomized as never smokers, former smoker, and current smokers (regular and occasional smokers) in this analysis. Alcohol intake was evaluated by participants self-reported

information on beverage-specific alcohol consumption (beer, wine, or spirits) on the last weekday and last weekend. Physical activity levels were based on self-reported time per week spent on physical activity during leisure time in summer and winter, and then were dichotomized as low physical activity (almost no activity), medium physical activity (regularly or irregularly about one hour per week), and high physical activity (regularly more than two hours per week) in present analysis.

Study participants without a diagnosis of diabetes underwent a standard oral glucose tolerance test (OGTT). Normal glucose tolerance was defined as a fasting glucose concentration of less than 110 mg/dL and a 2-hour glucose concentration of less than 140 mg/dL. Prediabetes was defined as a fasting glucose concentration of 110 -125 mg/dL, a 2-hour glucose concentration of 140-199 mg/dL, or a combination of both. Diabetes was defined as a fasting glucose concentration ≥ 126 mg/dL or a 2-hour glucose concentration ≥ 200 mg/dL during the OGTT, self-reported diagnosis of diabetes, or use of antidiabetic medication. Hypertension was defined using self-reported use of antihypertensive medication or blood pressure measurements (over 140/90 mm Hg). Angina pectoris was defined using self-reported history of physician diagnoses. Stroke or myocardial infarction was defined using self-reported history of physician diagnoses treated in hospital.

Medication data were collected using IDOM software (an instrument for database-supported online medication registration)[3], and included antihypertensive medication (antihypertensive medication, beta-blocker, diuretics, ACE inhibitors, calcium antagonists) and nonsteroidal anti-inflammatory drugs (NASID).

**Table S1. Biomarkers of subclinical inflammation in the OLINK Inflammation panel and assay characteristics.**

| Assay ID | Biomarker | Full name | UniProt No | Gene symbol | Intra-assay CV (%) | Inter-assay CV (%) | LOD (NPX) | Percentage of samples below LOD |
|---|---|---|---|---|---|---|---|---|
| 101_IL-8 | IL-8 | Interleukin-8 | P10145 | CXCL8 | 3 | 8.9 | 1.69 | 0 |
| 102_VEGF-A | VEGF-A | Vascular endothelial growth factor A | P15692 | VEGFA | 2.9 | 7.6 | 2.22 | 0 |
| 103_BDNF* | BDNF | Brain-derived neurotrophic factor | P23560 | BDNF | 2.8 | 8.1 | 0.09 | 26.9 |
| 105_MCP-3 | MCP-3 | Monocyte chemotactic protein 3 (CCL7) | P80098 | CCL7 | 6.5 | 10.3 | 0.84 | 3 |
| 106_GDNF | GDNF | Glial cell line-derived neurotrophic factor | P39905 | GDNF | 8.5 | 8.4 | 0.7 | 9.5 |
| 107_CDCP1 | CDCP1 | CUB domain-containing protein 1 | Q9H5V8 | CDCP1 | 3.9 | 9.6 | 0.11 | 0 |
| 108_CD244 | CD244 | Natural killer cell receptor 2B4 | Q9BZW8 | CD244 | 2.2 | 8.9 | 0.66 | 0 |
| 109_IL-7 | IL-7 | Interleukin-7 | P13232 | IL7 | 2.7 | 6 | 1.04 | 0.1 |
| 110_OPG | OPG | Osteoprotegerin | O00300 | TNFRSF11B | 2.3 | 5 | 0.85 | 0 |
| 111_LAP TGF-beta-1 | LAP TGFβ1 | Latency-associated peptide transforming growth factor beta-1 | P01137 | TGFB1 | 10 | 16.6 | 0.61 | 0.1 |
| 112_uPA | uPA | Urokinase-type plasminogen activator | P00749 | PLAU | 2.5 | 4.6 | 0.81 | 0 |
| 113_IL-6 | IL-6 | Interleukin-6 | P05231 | IL6 | 4.4 | 10.7 | 1.15 | 2.1 |

| | | | | | | | | |
|---|---|---|---|---|---|---|---|---|
| 114_IL-17C | IL-17C | Interleukin-17C | Q9P0M4 | IL17C | 6.2 | 6.9 | 0.95 | 22.3 |
| 115_MCP-1 | MCP-1 | Monocyte chemotactic protein 1 (CCL2) | P13500 | CCL2 | 2.9 | 6 | 1.25 | 0 |
| 116_IL-17A* | IL-17A | Interleukin-17A | Q16552 | IL17A | 4.9 | 9.3 | 0.74 | 33 |
| 117_CXCL11 | CXCL11 | C-X-C motif chemokine 11 | O14625 | CXCL11 | 2.5 | 5.8 | 1.02 | 0 |
| 118_AXIN1 | Axin-1 | Axin-1 | O15169 | AXIN1 | 4.5 | 8.7 | 0.95 | 10.1 |
| 120_TRAIL | TRAIL | TNF-related apoptosis-inducing ligand (TNFSF10) | P50591 | TNFSF10 | 2.7 | 7.2 | 0.59 | 0 |
| 121_IL-20RA* | IL-20RA | Interleukin-20 receptor subunit alpha | Q9UHF4 | IL20RA | n/a | n/a | 0.61 | 93 |
| 122_CXCL9 | CXCL9 | C-X-C motif chemokine 9 | Q07325 | CXCL9 | 3.2 | 5.4 | 0.7 | 0 |
| 123_CST5 | CST5 | Cystatin D | P28325 | CST5 | 2.4 | 6.6 | 0.97 | 0 |
| 124_IL-2RB* | IL-2RB | Interleukin-2 receptor subunit beta | P14784 | IL2RB | n/a | n/a | 1.09 | 95.4 |
| 125_IL-1 alpha* | IL-1α | Interleukin-1 alpha | P01583 | IL1A | n/a | n/a | 1.77 | 96.9 |
| 126_OSM | OSM | Oncostatin-M | P13725 | OSM | 2.3 | 6.9 | 1.55 | 0.4 |
| 127_IL-2* | IL-2 | Interleukin-2 | P60568 | IL2 | n/a | n/a | 0.68 | 99.9 |
| 128_CXCL1 | CXCL1 | C-X-C motif chemokine 1 | P09341 | CXCL1 | 2.5 | 5.8 | 1.73 | 0 |
| 129_TSLP* | TSLP | Thymic stromal lymphopoietin | Q969D9 | TSLP | n/a | n/a | 1.41 | 99.2 |
| 130_CCL4 | CCL4 | C-C motif chemokine 4 | P13236 | CCL4 | 2.8 | 5.8 | 1.38 | 0 |
| 131_CD6 | CD6 | T cell surface glycoprotein CD6 isoform | Q8WWJ7 | CD6 | 3.8 | 12.9 | 1.55 | 0 |
| 132_SCF | SCF | Stem cell factor (c-Kit-ligand) | P21583 | KITLG | 2.1 | 5.8 | 1.08 | 0 |

| | | | | | | | | |
|---|---|---|---|---|---|---|---|---|
| 133_IL-18 | IL-18 | Interleukin-18 | Q14116 | IL18 | 3 | 6.4 | 1.36 | 0 |
| 134_SLAMF1 | SLAMF1 | Signaling lymphocytic activation molecule (SLAM) | Q13291 | SLAMF1 | 6.4 | 10.7 | 1.08 | 0 |
| 135_TGF-alpha | TGF-α | Transforming growth factor alpha | P01135 | TGFA | 3.1 | 9.5 | -0.19 | 0 |
| 136_MCP-4 | MCP-4 | Monocyte chemotactic protein 4 (CCL13) | Q99616 | CCL13 | 3 | 9.5 | 0.29 | 0 |
| 137_CCL11 | CCL11 | Eotaxin (CCL11) | P51671 | CCL11 | 2.8 | 6.9 | 1.43 | 0 |
| 138_TNFSF14 | TNFSF14 | Tumor necrosis factor ligand superfamily member 14 (LIGHT) | O43557 | TNFSF14 | 2.8 | 7.8 | 1.3 | 0 |
| 139_FGF-23 | FGF-23 | Fibroblast growth factor 23 | Q9GZV9 | FGF23 | 5 | 7.6 | 0.31 | 0.2 |
| 140_IL-10RA* | IL-10RA | Interleukin-10 receptor subunit alpha | Q13651 | IL10RA | 2.6 | 9.2 | 0.82 | 26.5 |
| 141_FGF-5 | FGF-5 | Fibroblast growth factor 5 | Q8NF90 | FGF5 | 4.3 | 8.7 | 0.61 | 2.5 |
| 142_MMP-1 | MMP-1 | Matrix metalloproteinase-1 | P03956 | MMP1 | 2.3 | 5.4 | 2.12 | 0 |
| 143_LIF-R | LIF-R | Leukemia inhibitory factor receptor | P42702 | LIFR | 3.4 | 10.1 | 1.2 | 0 |
| 144_FGF-21 | FGF-21 | Fibroblast growth factor 21 | Q9NSA1 | FGF21 | 3.1 | 7.3 | 0.84 | 0 |
| 145_CCL19 | CCL19 | C-C motif chemokine 19 | Q99731 | CCL19 | 2.9 | 7 | 0.9 | 0 |
| 148_IL-15RA | IL-15RA | Interleukin-15 receptor subunit alpha | Q13261 | IL15RA | 5.6 | 10.5 | 0.15 | 2.4 |
| 149_IL-10RB | IL-10RB | Interleukin-10 receptor subunit beta | Q08334 | IL10RB | 3.1 | 10.4 | 1.17 | 0 |

| | | | | | | | | |
|---|---|---|---|---|---|---|---|---|
| 150_IL-22 RA1* | IL-22RA1 | Interleukin-22 receptor subunit alpha-1 | Q8N6P7 | IL22RA1 | n/a | n/a | 1.55 | 99.8 |
| 151_IL-18R1 | IL-18R1 | Interleukin-18 receptor 1 | Q13478 | IL18R1 | 3 | 7.6 | 0.85 | 0 |
| 152_PD-L1 | PD-L1 | Programmed cell death 1 ligand 1 | Q9NZQ7 | CD274 | 4.8 | 9.6 | 1.87 | 0 |
| 153_Beta-NGF | Beta-NGF | Beta-nerve growth factor | P01138 | NGF | 3.6 | 7.6 | 1.02 | 0 |
| 154_CXCL5 | CXCL5 | C-X-C motif chemokine 5 | P42830 | CXCL5 | 2.7 | 5.8 | 1.57 | 0 |
| 155_TRANCE | TRANCE | TNF-related activation-induced cytokine (TRANCE, TNFSF11, RANKL, OPGL) | O14788 | TNFSF11 | 4.6 | 8.8 | 1.32 | 0 |
| 156_HGF | HGF | Hepatocyte growth factor | P14210 | HGF | 2.5 | 7.5 | 1.11 | 0 |
| 157_IL-12B | IL-12B | Interleukin-12 subunit beta | P29460 | IL12B | 3.2 | 6.9 | 0.69 | 0 |
| 158_IL-24* | IL-24 | Interleukin-24 | Q13007 | IL24 | n/a | n/a | 1.36 | 93.5 |
| 159_IL-13* | IL-13 | Interleukin-13 | P35225 | IL13 | n/a | n/a | 1.14 | 94.9 |
| 160_ARTN* | Artemin | Artemin | Q5T4W7 | ARTN | n/a | n/a | 0.72 | 95.3 |
| 161_MMP-10 | MMP-10 | Matrix metalloproteinase-10 (SL-2) | P09238 | MMP10 | 2.7 | 8.8 | 1.13 | 0 |
| 162_IL-10 | IL-10 | Interleukin-10 | P22301 | IL10 | 5.3 | 10.4 | 1.04 | 0 |
| 163_TNF* | TNFα | Tumor necrosis factor-alpha | P01375 | TNF | n/a | n/a | 1.08 | 95.9 |
| 164_CCL23 | CCL23 | C-C motif chemokine 23 | P55773 | CCL23 | 2.9 | 6.1 | 0.9 | 0 |
| 165_CD5 | CD5 | T-cell surface glycoprotein CD5 | P06127 | CD5 | 3.1 | 9.3 | 1.22 | 0 |

| | | | | | | | | |
|---|---|---|---|---|---|---|---|---|
| 166_MIP-1 alpha | MIP-1α | Macrophage inflammatory protein-1alpha (C-C motif chemokine 3/CCL3) | P10147 | CCL3 | 3.3 | 9 | 1.62 | 0 |
| 167_Flt3L | Flt3L | Fms-related tyrosine kinase 3 ligand | P49771 | FLT3LG | 2.7 | 8.1 | 1.28 | 0 |
| 168_CXCL6 | CXCL6 | C-X-C motif chemokine 6 | P80162 | CXCL6 | 2.4 | 8.9 | 1.26 | 0 |
| 169_CXCL10 | CXCL10 | C-X-C motif chemokine 10 (IP-10) | P02778 | CXCL10 | 3.1 | 6 | 1.53 | 0 |
| 170_4E-BP1 | EIF4EBP1 | Eukaryotic translation initiation factor 4E-binding protein 1 | Q13541 | EIF4EBP1 | 3.8 | 8.6 | 0.66 | 0 |
| 171_IL-20* | IL-20 | Interleukin-20 | Q9NYY1 | IL20 | n/a | n/a | 0.96 | 95.4 |
| 172_SIRT2 | SIRT2 | SIR2-like protein 2 | Q8IXJ6 | SIRT2 | 5.4 | 9.7 | 0.87 | 0 |
| 173_CCL28 | CCL28 | C-C motif chemokine 28 | Q9NRJ3 | CCL28 | 4.1 | 14.6 | 0.73 | 1 |
| 174_DNER | DNER | Delta and Notch-like epidermal growth factor-related receptor | Q8NFT8 | DNER | 2.3 | 8 | 0.71 | 0 |
| 175_EN-RAGE | EN-RAGE | Protein S100-A12 (EN-RAGE) | P80511 | S100A12 | 4.9 | 11 | 0.78 | 0 |
| 176_CD40 | CD40 | CD40L receptor | P25942 | CD40 | 2.5 | 7.8 | 0.81 | 0 |
| 177_IL-33* | IL-33 | Interleukin-33 | O95760 | IL33 | n/a | n/a | 0.84 | 98 |
| 178_IFN-gamma* | IFNγ | Interferon-gamma | P01579 | IFNG | n/a | n/a | 0.94 | 98.5 |
| 179_FGF-19 | FGF-19 | Fibroblast growth factor 19 | O95750 | FGF19 | 2.9 | 8.4 | 0.68 | 0 |
| 180_IL-4* | IL-4 | Interleukin-4 | P05112 | IL4 | n/a | n/a | 0.81 | 91.9 |

| | | | | | | | | |
|---|---|---|---|---|---|---|---|---|
| 181_LIF* | LIF | Leukemia inhibitory factor | P15018 | LIF | n/a | n/a | 1.28 | 95 |
| 182_NRTN* | Neurturin | Neurturin | Q99748 | NRTN | n/a | n/a | 1.04 | 97.2 |
| 183_MCP-2 | MCP-2 | Monocyte chemotactic protein 2 (MCP-2, CCL8) | P80075 | CCL8 | 2.7 | 8 | 1.27 | 0 |
| 184_CASP-8** | Caspase-8 | Caspase-8 | Q14790 | CASP8 | 7.1 | 35.9 | 1.46 | 0.7 |
| 185_CCL25 | CCL25 | C-C motif chemokine 25 | O15444 | CCL25 | 3.2 | 7.6 | 0.7 | 0 |
| 186_CX3CL1 | CX3CL1 | Fractalkine | P78423 | CX3CL1 | 3.7 | 10.8 | 1.31 | 0 |
| 187_TNFRSF9 | TNFRSF9 | Tumor necrosis factor receptor superfamily member 9 | Q07011 | TNFRSF9 | 3 | 9.7 | 1.31 | 0 |
| 188_NT-3 | NT-3 | Neurotrophin-3 | P20783 | NTF3 | 4.8 | 10.5 | 0.33 | 0.7 |
| 189_TWEAK | TWEAK | Tumor necrosis factor (Ligand) superfamily, member 12 (TWEAK) | O43508 | TNFSF12 | 2.3 | 6.2 | 1.07 | 0 |
| 190_CCL20 | CCL20 | C-C motif chemokine 20 | P78556 | CCL20 | 3.2 | 7.1 | 0.89 | 0 |
| 191_ST1A1 | ST1A1 | Sulfotransferase 1A1 | P50225 | SULT1A1 | 6.4 | 14 | 0.69 | 4.5 |
| 192_STAMPB | STAMBP | STAM-binding protein | O95630 | STAMBP | 3.6 | 9.2 | 1.16 | 0 |
| 193_IL-5* | IL-5 | Interleukin-5 | P05113 | IL5 | n/a | n/a | 1.21 | 72.6 |
| 194_ADA | ADA | Adenosine deaminase | P00813 | ADA | 3.7 | 8.8 | 0.99 | 0 |
| 195_TNFB | TNFβ | Tumor necrosis factor-beta (lymphotoxin-alpha/LT-alpha) | P01374 | LTA | 4.4 | 8.8 | 1.08 | 0 |
| 196_CSF-1 | CSF-1 | Macrophage colony-stimulating factor 1 | P09603 | CSF1 | 2.2 | 8.8 | 0.93 | 0 |

Note: LOD, limit of detection; n/a, not applicable (NPX of control measurements below LOD); NPX, normalized protein expression values; *Data missing for more than 25% were excluded from analysis; **Data for inter-assay CVs >20% were excluded from analysis.

**Table S2. Levels of 71 biomarkers of subclinical inflammation in serum.**

| Biomarkers (NPX) | Groups | Mean ± SD | 25% | Median | 75% |
|---|---|---|---|---|---|
| ADA | Enzymes | 3.8 ± 0.4 | 3.6 | 3.8 | 4.0 |
| Axin-1 | Miscellaneous | 1.6 ± 0.5 | 1.2 | 1.5 | 1.9 |
| Beta-NGF | Neurotrophic factors | 1.9 ± 0.3 | 1.7 | 1.8 | 2.0 |
| CCL11 | Chemokines | 8.7 ± 0.4 | 8.4 | 8.7 | 9.0 |
| CCL19 | Chemokines | 10.0 ± 1.0 | 9.3 | 9.8 | 10.4 |
| CCL20 | Chemokines | 4.9 ± 1.0 | 4.2 | 4.8 | 5.5 |
| CCL23 | Chemokines | 10 ± 0.5 | 9.7 | 10.0 | 10.3 |
| CCL25 | Chemokines | 6.7 ± 0.6 | 6.3 | 6.7 | 7.0 |
| CCL28 | Chemokines | 1.6 ± 0.4 | 1.3 | 1.6 | 1.8 |
| CCL4 | Chemokines | 8.2 ± 0.6 | 7.9 | 8.2 | 8.6 |
| CD244 | Miscellaneous | 5.6 ± 0.3 | 5.3 | 5.6 | 5.8 |
| CD40 | TNF(R) superfamily | 10.3 ± 0.3 | 10.1 | 10.3 | 10.5 |
| CD5 | Miscellaneous | 5.4 ± 0.4 | 5.1 | 5.4 | 5.6 |
| CD6 | Miscellaneous | 4.6 ± 0.5 | 4.3 | 4.6 | 4.9 |
| CDCP1 | Miscellaneous | 3.4 ± 0.7 | 3.0 | 3.3 | 3.8 |
| CSF-1 | Cytokines/growth factors | 7.8 ± 0.2 | 7.6 | 7.8 | 8.0 |
| CST5 | Miscellaneous | 6.5 ± 0.5 | 6.1 | 6.4 | 6.8 |
| CX3CL1 | Chemokines | 6.3 ± 0.4 | 6.0 | 6.3 | 6.5 |
| CXCL1 | Chemokines | 9.4 ± 0.5 | 9.1 | 9.4 | 9.7 |
| CXCL10 | Chemokines | 9.5 ± 0.8 | 9.0 | 9.4 | 9.9 |
| CXCL11 | Chemokines | 7.9 ± 0.7 | 7.5 | 7.9 | 8.3 |

| | | | | | |
|---|---|---|---|---|---|
| CXCL5 | Chemokines | 11.5 ± 0.7 | 11.1 | 11.6 | 12.1 |
| CXCL6 | Chemokines | 8.7 ± 0.6 | 8.3 | 8.7 | 9.1 |
| CXCL9 | Chemokines | 7.5 ± 0.8 | 6.9 | 7.4 | 8.0 |
| DNER | Miscellaneous | 8.3 ± 0.3 | 8.1 | 8.3 | 8.5 |
| EIF4EBP1 | Miscellaneous | 7.0 ± 0.6 | 6.6 | 7.0 | 7.4 |
| EN-RAGE | Cytokines/growth factors | 5.1 ± 0.8 | 4.5 | 5.0 | 5.6 |
| FGF-19 | Cytokines/growth factors | 7.8 ± 0.9 | 7.2 | 7.8 | 8.4 |
| FGF-21 | Cytokines/growth factors | 6.0 ± 1.1 | 5.3 | 5.9 | 6.6 |
| FGF-23 | Cytokines/growth factors | 1.4 ± 0.5 | 1.1 | 1.4 | 1.7 |
| FGF-5 | Cytokines/growth factors | 1.3 ± 0.3 | 1.1 | 1.3 | 1.5 |
| Flt3L | Cytokines/growth factors | 9.0 ± 0.4 | 8.7 | 9.0 | 9.2 |
| GDNF | Neurotrophic factors | 1.1 ± 0.3 | 0.9 | 1.1 | 1.3 |
| HGF | Cytokines/growth factors | 8.6 ± 0.4 | 8.4 | 8.6 | 8.9 |
| IL-10 | Interleukins (receptors) | 2.5 ± 0.4 | 2.2 | 2.5 | 2.7 |
| IL-10RB | Interleukins (receptors) | 7.3 ± 0.3 | 7.1 | 7.3 | 7.5 |
| IL-12B | Interleukins (receptors) | 5.5 ± 0.7 | 5.0 | 5.4 | 5.9 |
| IL-15RA | Interleukins (receptors) | 0.8 ± 0.3 | 0.6 | 0.8 | 1.0 |
| IL-17C | Interleukins (receptors) | 1.4 ± 0.5 | 1.0 | 1.3 | 1.7 |
| IL-18 | Interleukins (receptors) | 8.7 ± 0.6 | 8.4 | 8.7 | 9.1 |
| IL-18R1 | Interleukins (receptors) | 7.5 ± 0.4 | 7.2 | 7.5 | 7.8 |
| IL-6 | Interleukins (receptors) | 2.4 ± 0.7 | 1.9 | 2.3 | 2.8 |
| IL-7 | Interleukins (receptors) | 4.7 ± 0.5 | 4.4 | 4.7 | 5.0 |
| IL-8 | Interleukins (receptors) | 6.7 ± 0.5 | 6.3 | 6.7 | 7.0 |

| | | | | | |
|---|---|---|---|---|---|
| LAP TGFβ1 | Cytokines/growth factors | 8.0 ± 0.3 | 7.7 | 8.0 | 8.2 |
| LIF-R | Miscellaneous | 4.7 ± 0.3 | 4.4 | 4.7 | 4.9 |
| MCP-1 | Chemokines | 11.1 ± 0.4 | 10.8 | 11.1 | 11.4 |
| MCP-2 | Chemokines | 9.5 ± 0.7 | 9.1 | 9.6 | 10 |
| MCP-3 | Chemokines | 2.0 ± 0.5 | 1.6 | 1.9 | 2.2 |
| MCP-4 | Chemokines | 4.1 ± 0.6 | 3.7 | 4.1 | 4.4 |
| MIP-1α | Chemokines | 4.5 ± 0.5 | 4.2 | 4.5 | 4.8 |
| MMP-1 | Enzymes | 14.5 ± 0.7 | 14 | 14.6 | 15.0 |
| MMP-10 | Enzymes | 6.4 ± 0.6 | 6.1 | 6.4 | 6.8 |
| NT-3 | Neurotrophic factors | 1.0 ± 0.3 | 0.8 | 1.0 | 1.2 |
| OPG | TNF(R) superfamily | 10.2 ± 0.3 | 10 | 10.2 | 10.5 |
| OSM | Cytokines/growth factors | 4.9 ± 0.6 | 4.5 | 4.9 | 5.3 |
| PD-L1 | Miscellaneous | 4.3 ± 0.4 | 4.1 | 4.3 | 4.5 |
| SCF | Cytokines/growth factors | 9.9 ± 0.4 | 9.7 | 10 | 10.2 |
| SIRT2 | Enzymes | 2.3 ± 0.5 | 2.0 | 2.3 | 2.6 |
| SLAMF1 | Miscellaneous | 2.5 ± 0.5 | 2.2 | 2.5 | 2.9 |
| ST1A1 | Enzymes | 2.0 ± 0.8 | 1.4 | 1.9 | 2.5 |
| STAMBP | Enzymes | 3.1 ± 0.4 | 2.8 | 3.0 | 3.3 |
| TGFα | Cytokines/growth factors | 4.6 ± 0.5 | 4.2 | 4.6 | 4.9 |
| TNFRSF9 | TNF(R) superfamily | 6.3 ± 0.5 | 6.0 | 6.3 | 6.6 |
| TNFSF14 | TNF(R) superfamily | 5.6 ± 0.5 | 5.3 | 5.6 | 6.0 |
| TNFβ | TNF(R) superfamily | 3.9 ± 0.5 | 3.6 | 3.9 | 4.1 |
| TRAIL | TNF(R) superfamily | 8.1 ± 0.3 | 7.9 | 8.1 | 8.3 |

| | | | | | |
|---|---|---|---|---|---|
| TRANCE | TNF(R) superfamily | 4.6 ± 0.6 | 4.2 | 4.6 | 5.0 |
| TWEAK | TNF(R) superfamily | 9.4 ± 0.3 | 9.2 | 9.4 | 9.6 |
| uPA | Enzymes | 9.9 ± 0.3 | 9.7 | 9.9 | 10.1 |
| VEGF-A | Cytokines/growth factors | 10.9 ± 0.5 | 10.6 | 10.9 | 11.3 |

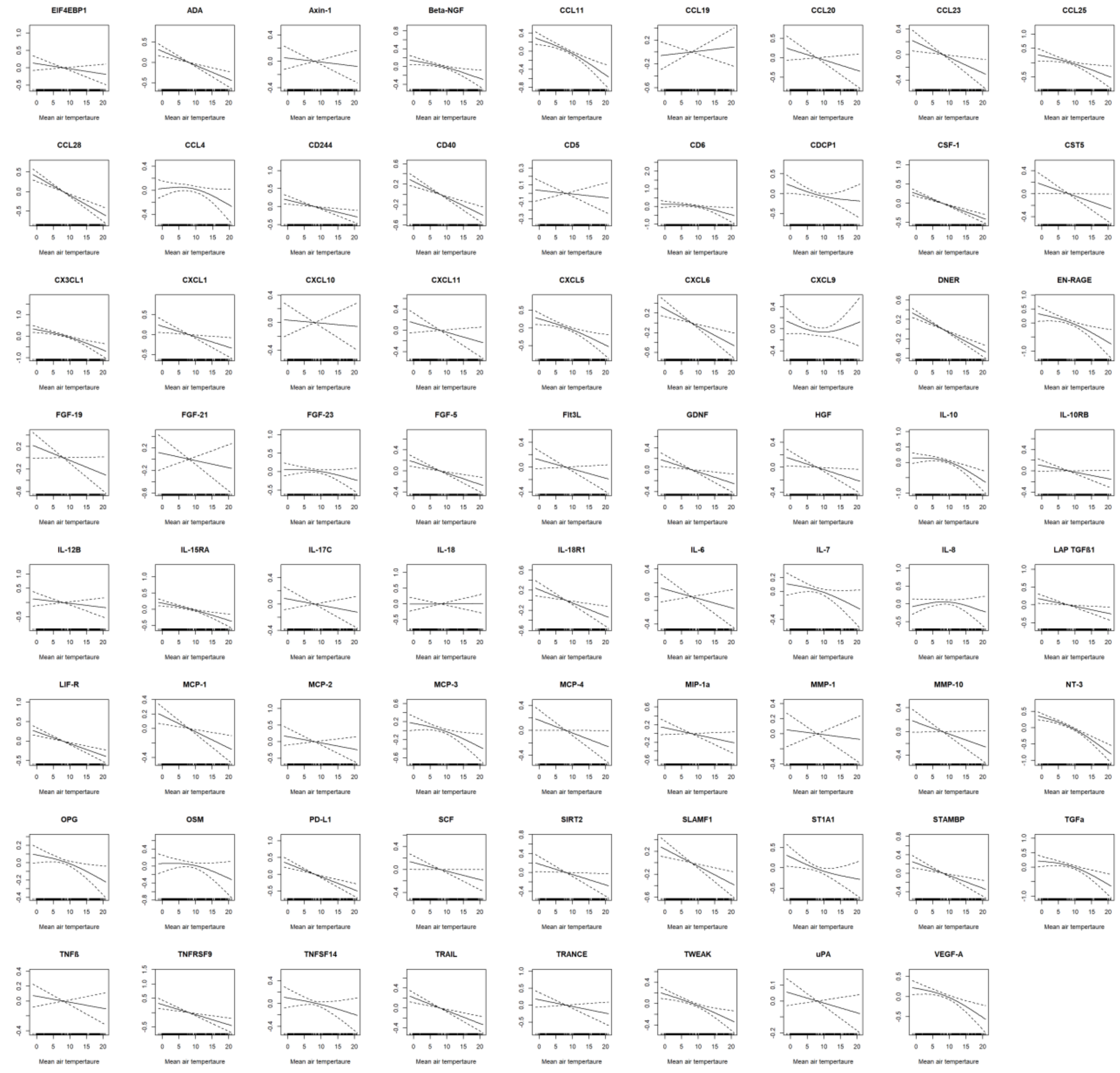


**Figure S1. Exposure-response functions of air temperature and biomarkers of subclinical inflammation at lags 0-13 days.**

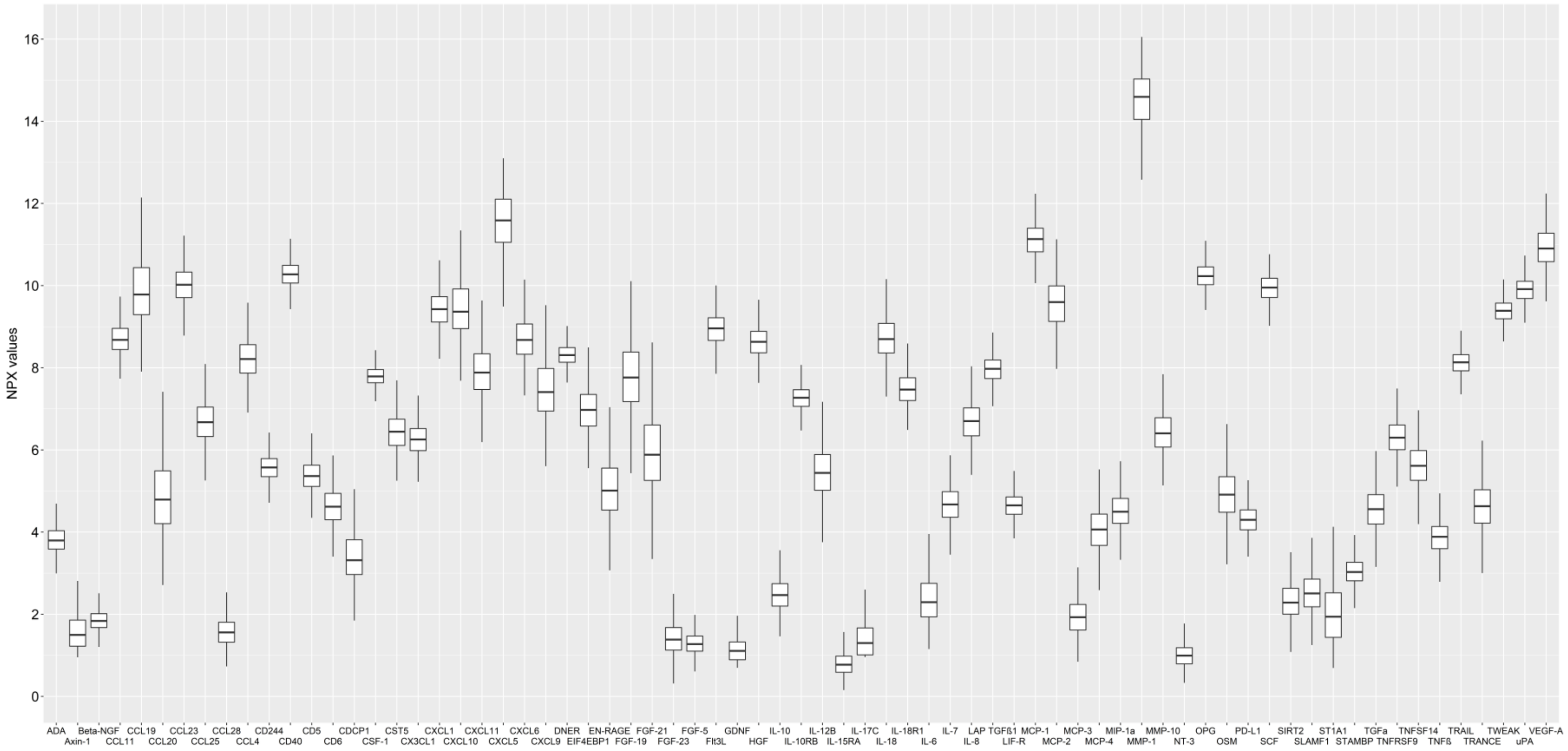


**Figure S2. The levels of 71 biomarkers of subclinical inflammation in serum samples.**

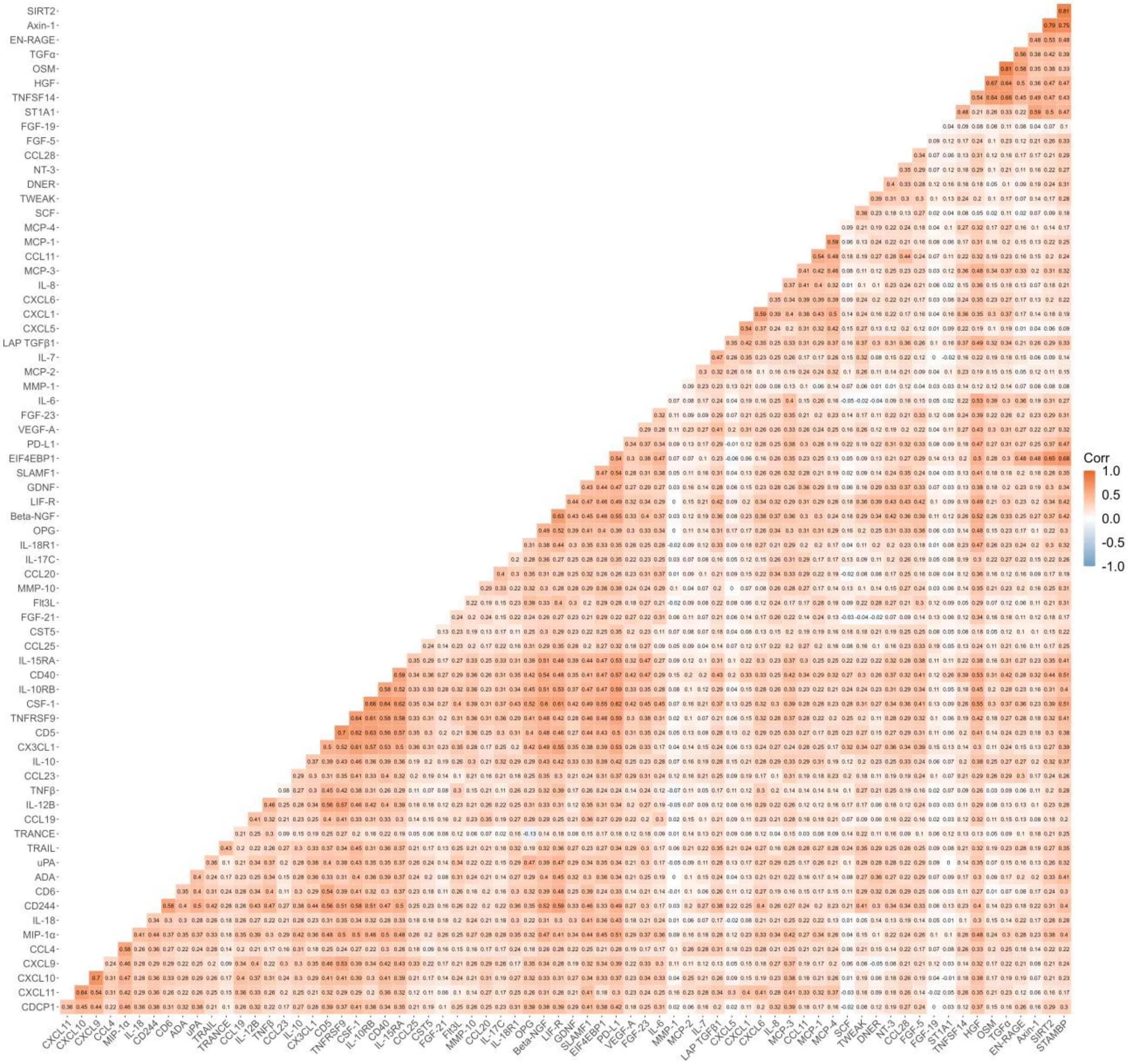


**Figure S3. Spearman correlation between biomarkers of subclinical inflammation.**

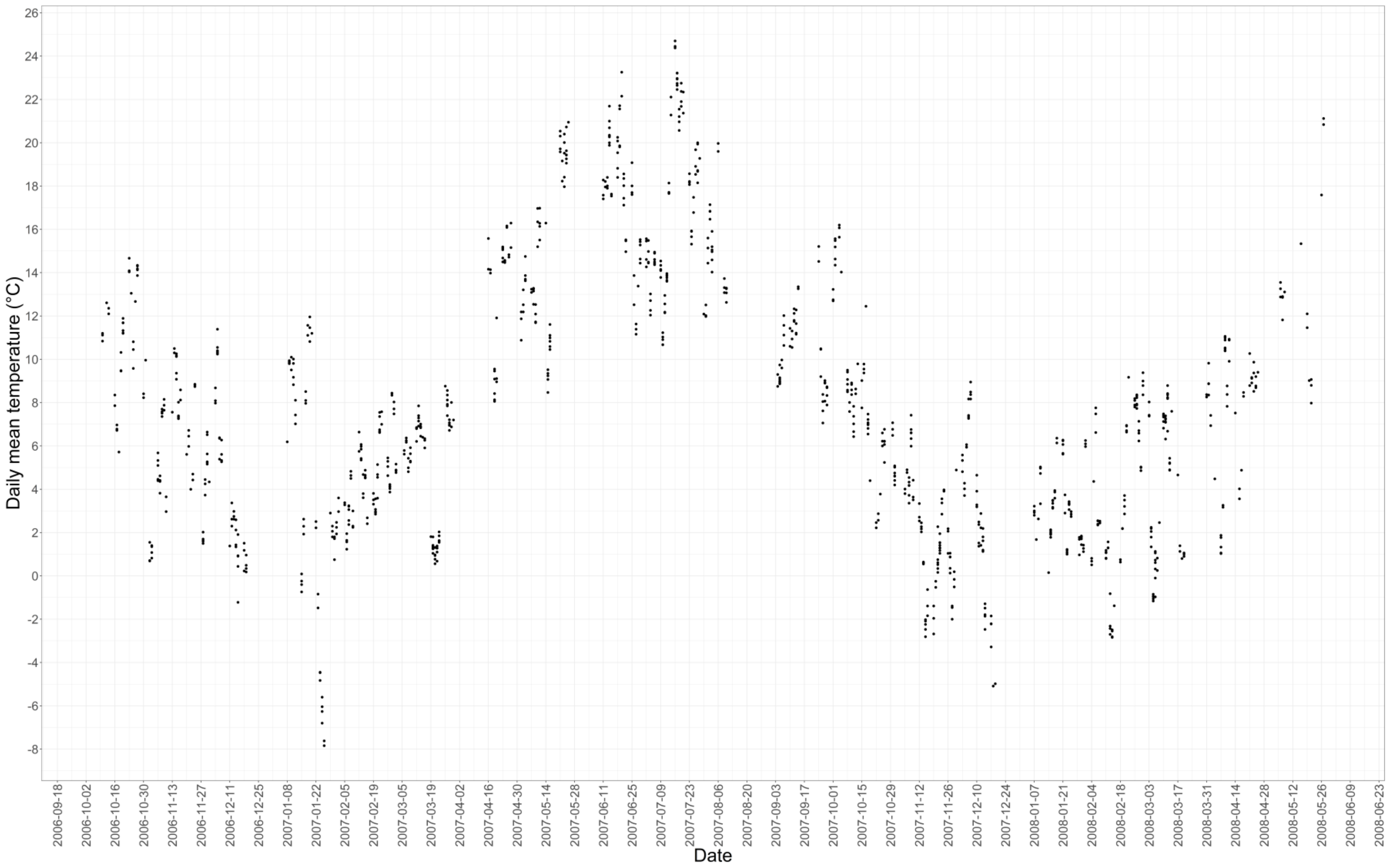


**Figure S4. Time series of daily mean air temperature for participants in this study.**

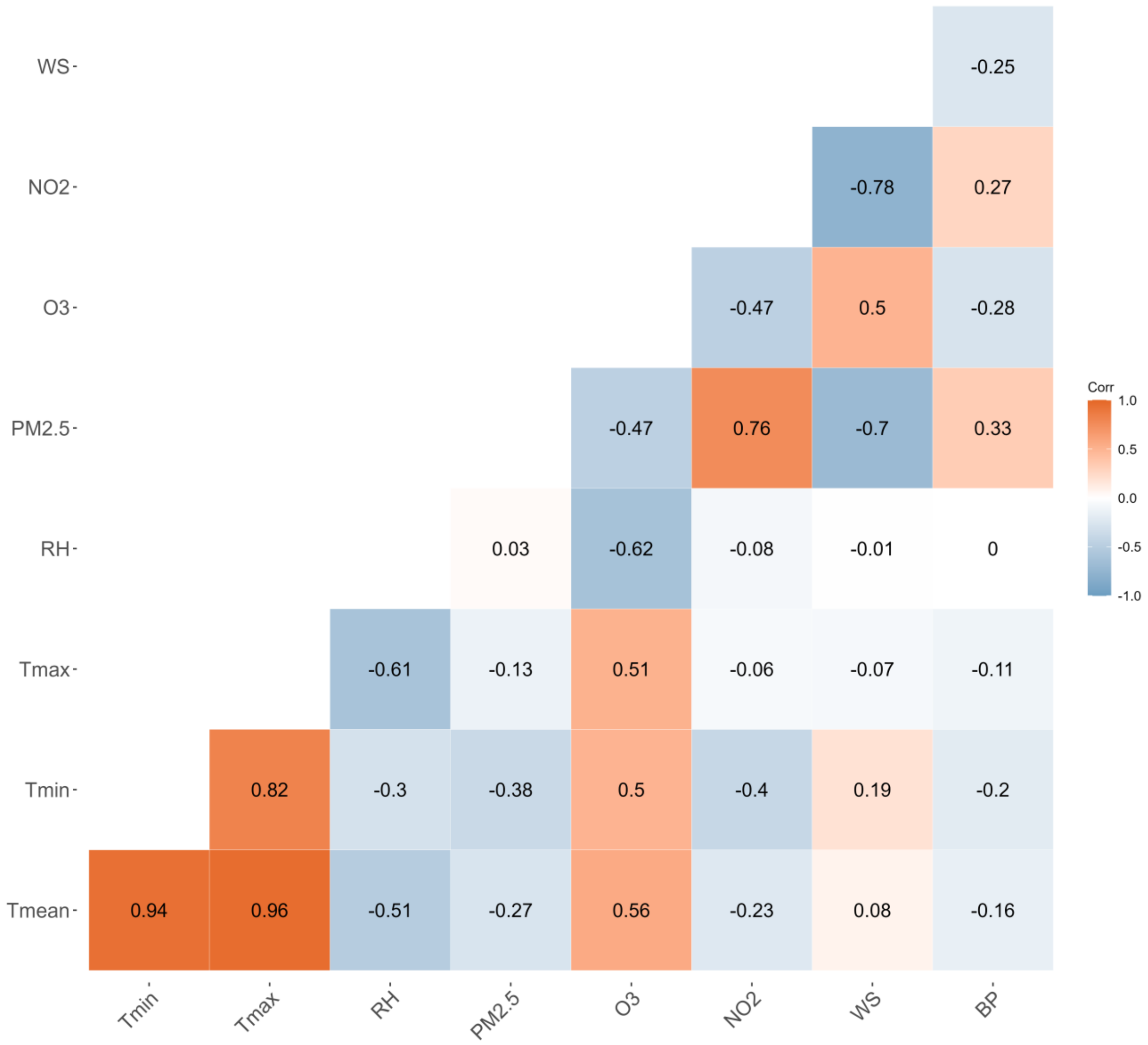


**Figure S5. Correlation between meteorological variables and air pollutants.**

Note: *Tmean***:** daily mean air temperature; *Tmin*: daily minimum air temperature; *Tmax*: daily maximum air temperature; *RH*: relative humidity; $O_3$: ozone; $PM_{2.5}$: particulate matter with an aerodynamic diameter of ≤2.5 μm; $NO_2$: nitrogen dioxide, *WS:* wind speed; *BP:* Barometric pressure.

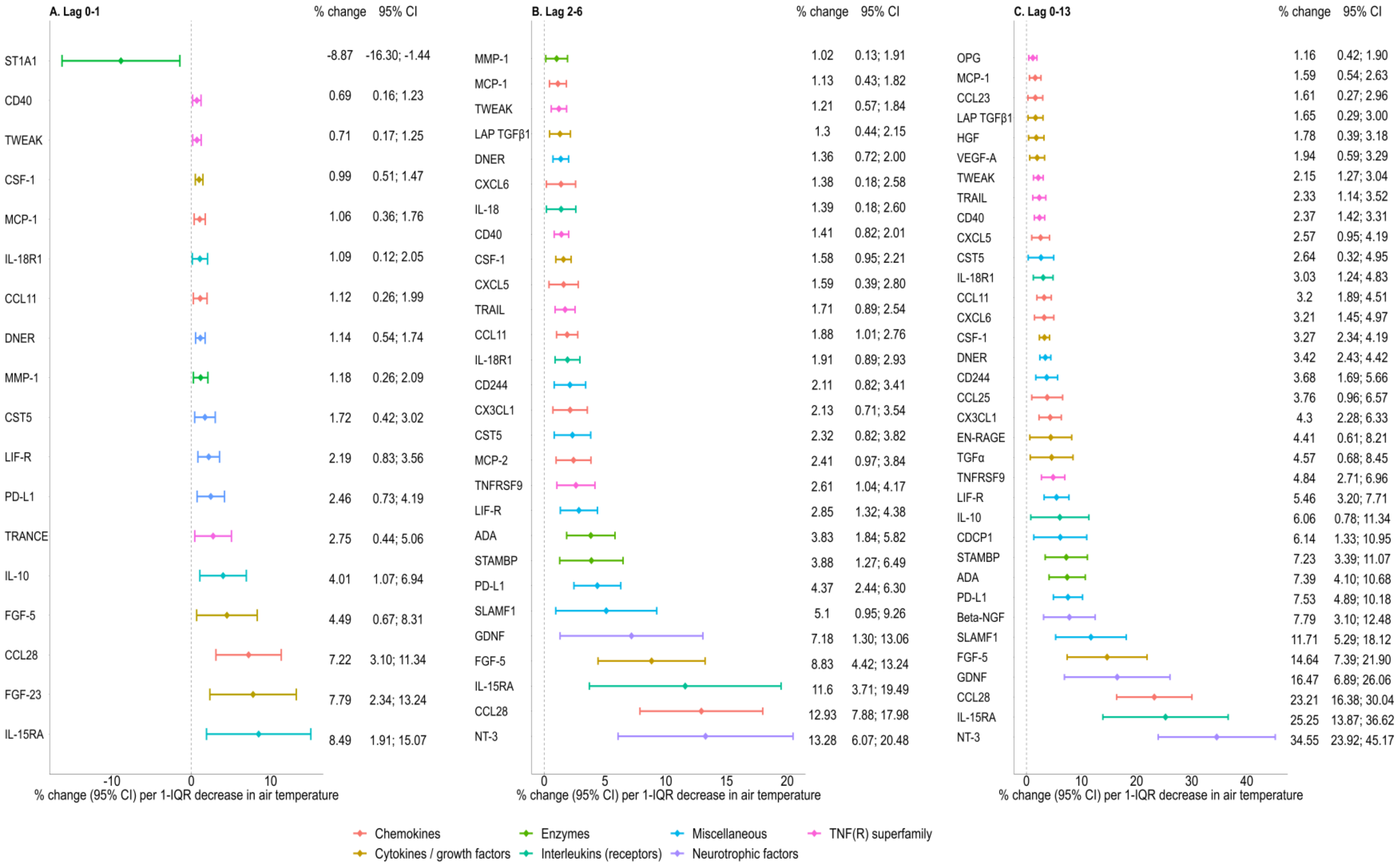
A. Lag 0-1
% change 95% CI
ST1A1 -8.87 -16.30; -1.44
CD40 0.69 0.16; 1.23
TWEAK 0.71 0.17; 1.25
CSF-1 0.99 0.51; 1.47
MCP-1 1.06 0.36; 1.76
IL-18R1 1.09 0.12; 2.05
CCL11 1.12 0.26; 1.99
DNER 1.14 0.54; 1.74
MMP-1 1.18 0.26; 2.09
CST5 1.72 0.42; 3.02
LIF-R 2.19 0.83; 3.56
PD-L1 2.46 0.73; 4.19
TRANCE 2.75 0.44; 5.06
IL-10 4.01 1.07; 6.94
FGF-5 4.49 0.67; 8.31
CCL28 7.22 3.10; 11.34
FGF-23 7.79 2.34; 13.24
IL-15RA 8.49 1.91; 15.07
-10 0 10
% change (95% CI) per 1-IQR decrease in air temperature
B. Lag 2-6
% change 95% CI
MMP-1 1.02 0.13; 1.91
MCP-1 1.13 0.43; 1.82
TWEAK 1.21 0.57; 1.84
LAP TGFβ1 1.3 0.44; 2.15
DNER 1.36 0.72; 2.00
CXCL6 1.38 0.18; 2.58
IL-18 1.39 0.18; 2.60
CD40 1.41 0.82; 2.01
CSF-1 1.58 0.95; 2.21
CXCL5 1.59 0.39; 2.80
TRAIL 1.71 0.89; 2.54
CCL11 1.88 1.01; 2.76
IL-18R1 1.91 0.89; 2.93
CD244 2.11 0.82; 3.41
CX3CL1 2.13 0.71; 3.54
CST5 2.32 0.82; 3.82
MCP-2 2.41 0.97; 3.84
TNFRSF9 2.61 1.04; 4.17
LIF-R 2.85 1.32; 4.38
ADA 3.83 1.84; 5.82
STAMBP 3.88 1.27; 6.49
PD-L1 4.37 2.44; 6.30
SLAMF1 5.1 0.95; 9.26
GDNF 7.18 1.30; 13.06
FGF-5 8.83 4.42; 13.24
IL-15RA 11.6 3.71; 19.49
CCL28 12.93 7.88; 17.98
NT-3 13.28 6.07; 20.48
0 5 10 15 20
% change (95% CI) per 1-IQR decrease in air temperature
C. Lag 0-13
% change 95% CI
OPG 1.16 0.42; 1.90
MCP-1 1.59 0.54; 2.63
CCL23 1.61 0.27; 2.96
LAP TGFβ1 1.65 0.29; 3.00
HGF 1.78 0.39; 3.18
VEGF-A 1.94 0.59; 3.29
TWEAK 2.15 1.27; 3.04
TRAIL 2.33 1.14; 3.52
CD40 2.37 1.42; 3.31
CXCL5 2.57 0.95; 4.19
CST5 2.64 0.32; 4.95
IL-18R1 3.03 1.24; 4.83
CCL11 3.2 1.89; 4.51
CXCL6 3.21 1.45; 4.97
CSF-1 3.27 2.34; 4.19
DNER 3.42 2.43; 4.42
CD244 3.68 1.69; 5.66
CCL25 3.76 0.96; 6.57
CX3CL1 4.3 2.28; 6.33
EN-RAGE 4.41 0.61; 8.21
TGFα 4.57 0.68; 8.45
TNFRSF9 4.84 2.71; 6.96
LIF-R 5.46 3.20; 7.71
IL-10 6.06 0.78; 11.34
CDCP1 6.14 1.33; 10.95
STAMBP 7.23 3.39; 11.07
ADA 7.39 4.10; 10.68
PD-L1 7.53 4.89; 10.18
Beta-NGF 7.79 3.10; 12.48
SLAMF1 11.71 5.29; 18.12
FGF-5 14.64 7.39; 21.90
GDNF 16.47 6.89; 26.06
CCL28 23.21 16.38; 30.04
IL-15RA 25.25 13.87; 36.62
NT-3 34.55 23.92; 45.17
0 10 20 30 40
% change (95% CI) per 1-IQR decrease in air temperature
Chemokines
Enzymes
Miscellaneous
TNF(R) superfamily
Cytokines / growth factors
Interleukins (receptors)
Neurotrophic factors

**Figure S6. Significant associations between short-term exposure to air temperature per 1-IQR decrease with biomarkers of subclinical inflammation (P-adjust <0.05).**

Note: 1-IQR decrease was 9.2°C for lags 0-1 days, 8.9°C for lags 2-6 days, 8.4°C for lags 0-13 days. *ADA:* Adenosine deaminase, *Beta-NGF*: Beta-nerve growth factor, *CCL11*: Eotaxin, *CCL23*: C-C motif chemokine 23, *CCL25*: C-C motif chemokine 25, *CCL28*: C-C motif chemokine 28, *CD244*: Natural killer cell receptor 2B4, *CD40*: CD40L receptor, *CDCP1*: CUB domain-containing protein 1, *CSF-1*: Macrophage colony-stimulating factor 1, *CST5*: Cystatin D, *CX3CL1*: Fractalkine, *CXCL5*: C-X-C motif chemokine 5, *CXCL6*: C-X-C motif chemokine 6, *DNER*: Delta and Notch-like epidermal growth factor-related receptor, *EN-RAGE*: Protein S100-A12, *FGF-23*: Fibroblast growth factor 23, *FGF-5*: Fibroblast growth factor 5, *GDNF*: Glial cell line-derived neurotrophic factor, *HGF*: Hepatocyte growth factor, *IL-10*: Interleukin-10, *IL-15RA*: Interleukin-15 receptor subunit alpha, *IL-18*: Interleukin-18, *IL-18R1*: Interleukin-18 receptor 1, *LAP TGFß1*: Latency-associated peptide transforming growth factor beta-1, *LIF-R*: Leukemia inhibitory factor receptor, *MCP-1*: Monocyte chemotactic protein 1, *MCP-2*: Monocyte chemotactic protein 2, *MMP-1*: Matrix metalloproteinase-1, *NT-3*: Neurotrophin-3, *OPG*: Osteoprotegerin, *PD-L1*: Programmed cell death 1 ligand 1, *SLAMF1*: Signaling lymphocytic activation molecule, *ST1A1*: Sulfotransferase 1A1, *STAMBP*: STAM-binding protein, *TGFα*: Transforming growth factor alpha, *TNFRSF9*: Tumor necrosis factor receptor superfamily member 9, *TRAIL*: TNF-related apoptosis-inducing ligand, *TRANCE*: TNF-related activation-induced cytokine, *TWEAK*: Tumor necrosis factor (Ligand) superfamily, member 1, *VEGF-A*: Vascular endothelial growth factor A.

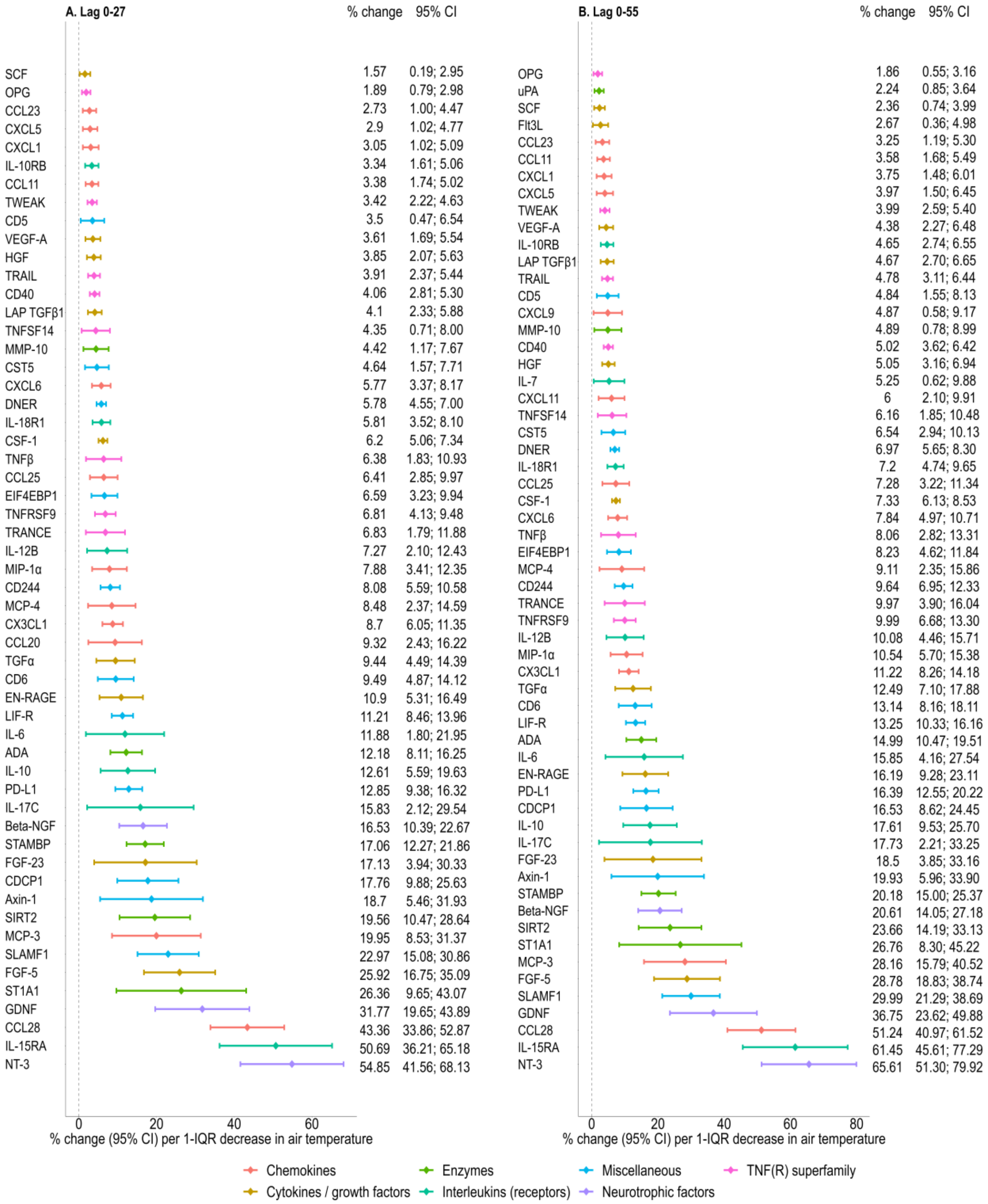


**Figure S7. Significant associations between medium-term exposure to air temperature per 1-IQR decrease with biomarkers of subclinical inflammation (*P*-adjust <0.05).**

Note: 1-IQR decrease was 9.0°C for lags 0-27 days, and 9.4°C for lags 0-55 days. *ADA:* Adenosine deaminase, *Beta-NGF*: Beta-nerve growth factor, *CCL11*: Eotaxin, *CCL20*: C-C motif chemokine

20, *CCL23*: C-C motif chemokine 23, *CCL25*: C-C motif chemokine 25, *CCL28*: C-C motif chemokine 28, *CD244*: Natural killer cell receptor 2B4, *CD40*: CD40L receptor, *CD5*: T-cell surface glycoprotein CD5, *CD6*: T cell surface glycoprotein CD6 isoform, *CDCP1*: CUB domain-containing protein 1, *CSF-1*: Macrophage colony-stimulating factor 1, *CST5*: Cystatin D, *CX3CL1*: Fractalkine, *CXCL1*: C-X-C motif chemokine 1, *CXCL11*: C-X-C motif chemokine 11, *CXCL5*: C-X-C motif chemokine 5, *CXCL6*: C-X-C motif chemokine 6, *CXCL9*: C-X-C motif chemokine 9, *DNER*: Delta and Notch-like epidermal growth factor-related receptor, *EIF4EBP1*: Eukaryotic translation initiation factor 4E-binding protein 1, *EN-RAGE*: Protein S100-A12, *FGF-23*: Fibroblast growth factor 23, *FGF-5*: Fibroblast growth factor 5, *Flt3L*: Fms-related tyrosine kinase 3 ligand, *GDNF*: Glial cell line-derived neurotrophic factor, *HGF*: Hepatocyte growth factor, *IL-10*: Interleukin-10, *IL-10RB*: Interleukin-10 receptor subunit beta, *IL-12B*: Interleukin-12 subunit beta, *IL-15RA*: Interleukin-15 receptor subunit alpha, *IL-17C*: Interleukin-17C, *IL-18R1*: Interleukin-18 receptor 1, *IL-6*: Interleukin-6, *IL-7*: Interleukin-7, *LAP TGFß1*: Latency-associated peptide transforming growth factor beta-1, *LIF-R*: Leukemia inhibitory factor receptor, *MCP-3*: Monocyte chemotactic protein 3, *MCP-4*: Monocyte chemotactic protein 4, *MIP-1α*: Macrophage inflammatory protein-1alpha, *MMP-10*: Matrix metalloproteinase-10, *NT-3*: Neurotrophin-3, *OPG*: Osteoprotegerin, *PD-L1*: Programmed cell death 1 ligand 1, *SCF*: Stem cell factor, *SIRT2*: SIR2-like protein 2, *SLAMF1*: Signaling lymphocytic activation molecule, *ST1A1*: Sulfotransferase 1A1, *STAMBP*: STAM-binding protein, *TGFα*: Transforming growth factor alpha, *TNFRSF9*: Tumor necrosis factor receptor superfamily member 9, *TNFß*: Tumor necrosis factor-beta, *TNFSF14*: Tumor necrosis factor ligand superfamily member 14, *TRAIL*: TNF-related apoptosis-inducing ligand, *TRANCE*: TNF-related activation-induced cytokine, *TWEAK*: Tumor necrosis factor (Ligand) superfamily, member 1, *uPA*: Urokinase-type plasminogen activator, *VEGF-A*: Vascular endothelial growth factor A.

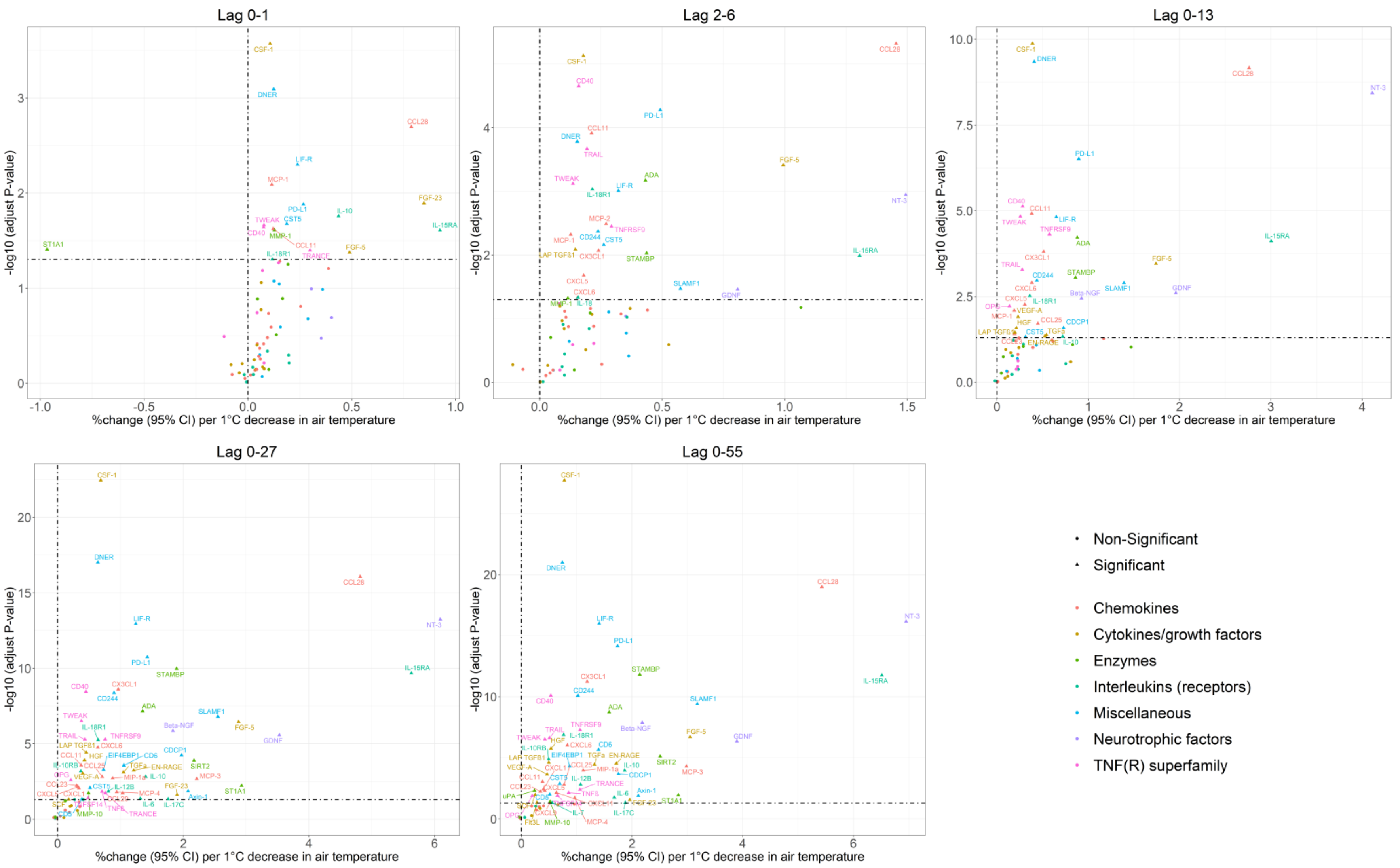

Lag 0-1
Lag 2-6
Lag 0-13
Lag 0-27
Lag 0-55
-log10 (adjust P-value)
%change (95% CI) per 1°C decrease in air temperature
Non-Significant
Significant
Chemokines
Cytokines/growth factors
Enzymes
Interleukins (receptors)
Miscellaneous
Neurotrophic factors
TNF(R) superfamily

**Figure S8. Volcano Plots presenting the associations between short- and medium-term exposures to air temperature per 1°C decrease with 71 biomarkers of subclinical inflammation.**

Note: 1-IQR decrease was 9.2°C for lags 0-1 days, 8.9°C for lags 2-6 days, 8.4°C for lags 0-13 days, 9.0°C for lags 0-27 days, and 9.4°C for lags 0-55 days. *ADA:* Adenosine deaminase, *Beta-NGF*: Beta-nerve growth factor, *CCL11*: Eotaxin, *CCL20*: C-C motif chemokine 20, *CCL23*: C-C motif chemokine 23, *CCL25*: C-C motif chemokine 25, *CCL28*: C-C motif chemokine 28, *CD244*: Natural killer cell receptor 2B4, *CD40*: CD40L receptor, *CD5*: T-cell surface glycoprotein CD5, *CD6*: T cell surface glycoprotein CD6 isoform, *CDCP1*: CUB domain-containing protein 1, *CSF-1*: Macrophage colony-stimulating factor 1, *CST5*: Cystatin D, *CX3CL1*: Fractalkine, *CXCL1*: C-X-C motif chemokine 1, *CXCL11*: C-X-C motif chemokine 11, *CXCL5*: C-X-C motif chemokine 5, *CXCL6*: C-X-C motif chemokine 6, *CXCL9*: C-X-C motif chemokine 9, *DNER*: Delta and Notch-like epidermal growth factor-related receptor, *EIF4EBP1*: Eukaryotic translation initiation factor 4E-binding protein 1, *EN-RAGE*: Protein S100-A12, *FGF-23*: Fibroblast growth factor 23, *FGF-5*: Fibroblast growth factor 5, *Flt3L*: Fms-related tyrosine kinase 3 ligand, *GDNF*: Glial cell line-derived neurotrophic factor, *HGF*: Hepatocyte growth factor, *IL-10*: Interleukin-10, *IL-10RB*: Interleukin-10 receptor subunit beta, *IL-12B*: Interleukin-12 subunit beta, *IL-15RA*: Interleukin-15 receptor subunit alpha, *IL-17C*: Interleukin-17C, *IL-18*: Interleukin-18, *IL-18R1*: Interleukin-18 receptor 1, *IL-6*: Interleukin-6, *IL-7*: Interleukin-7, *LAP TGFß1*: Latency-associated peptide transforming growth factor beta-1, *LIF-R*: Leukemia inhibitory factor receptor, *MCP-1*: Monocyte chemotactic protein 1, *MCP-2*: Monocyte chemotactic protein 2, *MCP-3*: Monocyte chemotactic protein 3, *MCP-4*: Monocyte chemotactic protein 4, *MIP-1α*: Macrophage inflammatory protein-1alpha, *MMP-1*: Matrix metalloproteinase-1, *MMP-10*: Matrix metalloproteinase-10, *NT-3*: Neurotrophin-3, *OPG*: Osteoprotegerin, *PD-L1*: Programmed cell death 1 ligand 1, *SCF*: Stem cell factor, *SIRT2*: SIR2-like protein 2, *SLAMF1*: Signaling lymphocytic activation molecule, *ST1A1*: Sulfotransferase 1A1, *STAMBP*: STAM-binding protein, *TGFα*: Transforming growth factor alpha, *TNFß*: Tumor necrosis factor-beta, *TNFRSF9*: Tumor necrosis factor receptor superfamily member 9, *TNFSF14*: Tumor necrosis factor ligand superfamily member 14, *TRAIL*: TNF-related apoptosis-inducing ligand, *TRANCE*: TNF-related activation-induced cytokine, *TWEAK*: Tumor

necrosis factor (Ligand) superfamily, member 1, *uPA*: Urokinase-type plasminogen activator, *VEGF-A*: Vascular endothelial growth factor A.

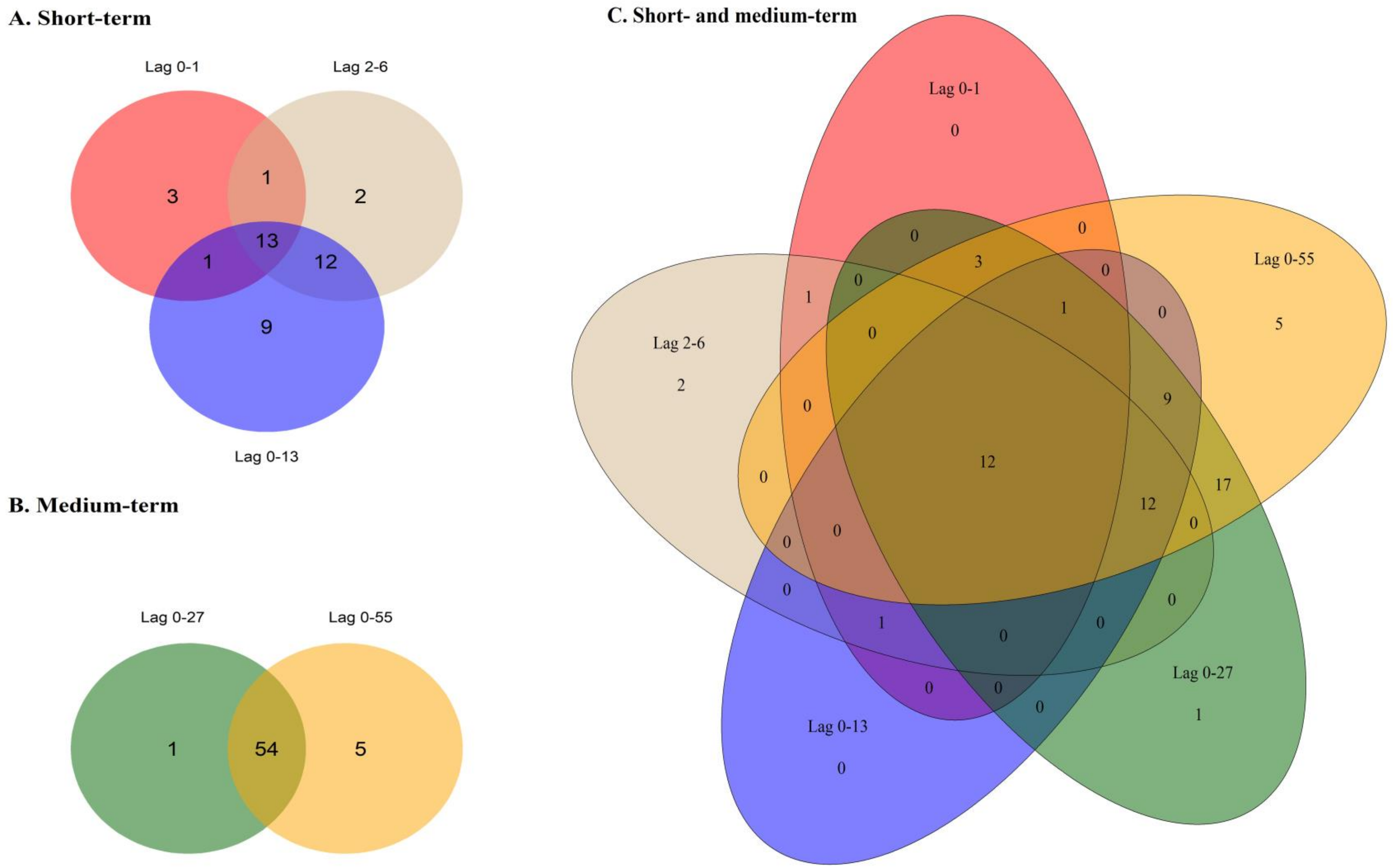


**Figure S9. Venn diagrams of significant associations between short- and medium-term exposures to air temperature per 1-IQR decrease with biomarkers of subclinical inflammation.**

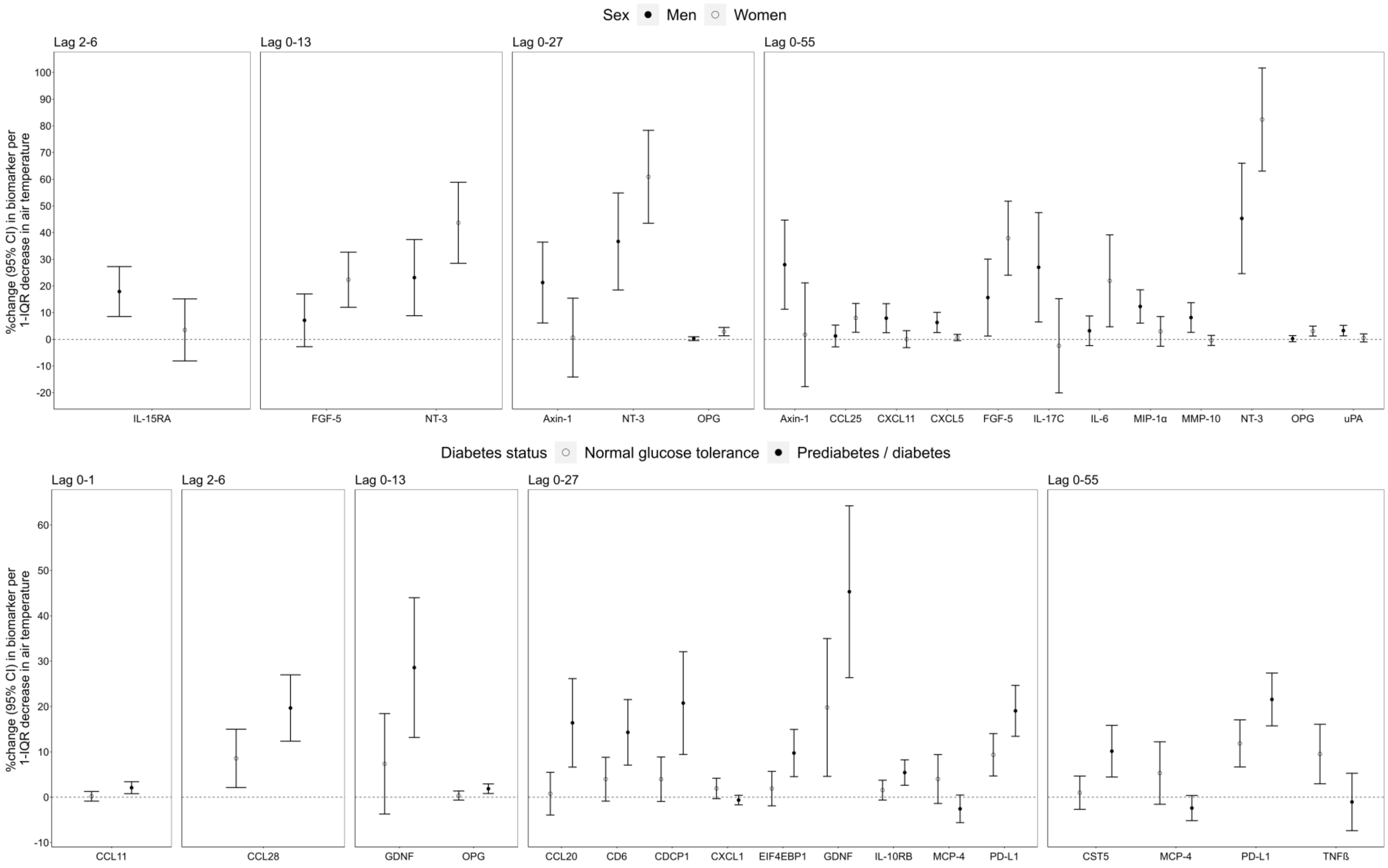

Sex Men Women
Lag 2-6
Lag 0-13
Lag 0-27
Lag 0-55
%change (95% CI) in biomarker per 1-IQR decrease in air temperature
IL-15RA
FGF-5
NT-3
Axin-1
NT-3
OPG
Axin-1
CCL25
CXCL11
CXCL5
FGF-5
IL-17C
IL-6
MIP-1α
MMP-10
NT-3
OPG
uPA
Diabetes status Normal glucose tolerance Prediabetes / diabetes
Lag 0-1
Lag 2-6
Lag 0-13
Lag 0-27
Lag 0-55
CCL11
CCL28
GDNF
OPG
CCL20
CD6
CDCP1
CXCL1
EIF4EBP1
GDNF
IL-10RB
MCP-4
PD-L1
CST5
MCP-4
PD-L1
TNFß

**Figure S10. Short- and medium-term effects of air temperature on biomarkers of subclinical inflammation per 1-IQR decrease significantly modified by sex or diabetes.**

Note: 1-IQR decrease was 9.2°C for lags 0-1 days, 8.9°C for lags 2-6 days, 8.4°C for lags 0-13 days, 9.0°C for lags 0-27 days, and 9.4°C for lags 0-55 days. *CCL11*: Eotaxin, *CCL20*: C-C motif chemokine 20, *CCL25*: C-C motif chemokine 25, *CCL28*: C-C motif chemokine 28, *CD6*: T cell surface glycoprotein CD6 isoform, *CDCP1*: CUB domain-containing protein 1, *CST5*: Cystatin D, *CX3CL1*: Fractalkine, *CXCL1*: C-X-C motif chemokine 1, *CXCL11*: C-X-C motif chemokine 11, *CXCL5*: C-X-C motif chemokine 5, *EIF4EBP1*: Eukaryotic translation initiation factor 4E-binding protein 1, *FGF-5*: Fibroblast growth factor 5, *GDNF*: Glial cell line-derived neurotrophic factor, *IL-10RB*: Interleukin-10 receptor subunit beta, *IL-15RA*: Interleukin-15 receptor subunit alpha, *IL-17C*: Interleukin-17C, *IL-6*: Interleukin-6, *MCP-4*: Monocyte chemotactic protein 4, *MIP-1α*: Macrophage inflammatory protein-1alpha, *MMP-10*: Matrix metalloproteinase-10, *NT-3*: Neurotrophin-3, *OPG*: Osteoprotegerin, *PD-L1*: Programmed cell death 1 ligand 1, *TNFß*: Tumor necrosis factor-beta, *uPA*: Urokinase-type plasminogen activator.

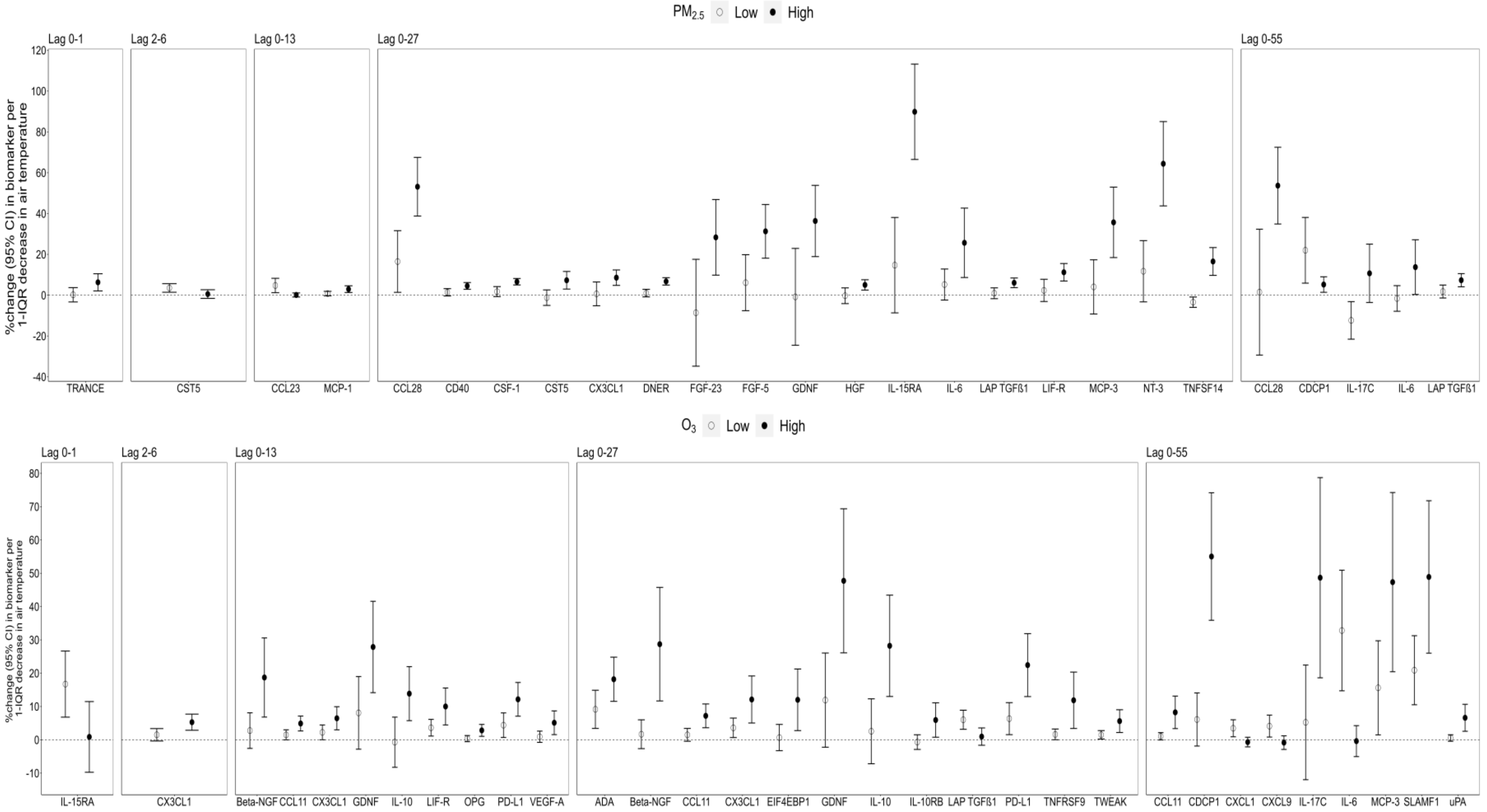


**Figure S11. Short- and medium-term effects of air temperature on biomarkers of subclinical inflammation per 1-IQR decrease significantly modified by $PM_{2.5}$ or $O_3$.**

Note: 1-IQR decrease was 9.2°C for lags 0-1 days, 8.9°C for lags 2-6 days, 8.4°C for lags 0-13 days, 9.0°C for lags 0-27 days, and 9.4°C for lags 0-55 days. *ADA:* Adenosine deaminase, *Beta-NGF*: Beta-nerve growth factor, *CCL11*: Eotaxin, *CCL23*: C-C motif chemokine

23, *CCL25*: C-C motif chemokine 25, *CCL28*: C-C motif chemokine 28, *CD40*: CD40L receptor, *CDCP1*: CUB domain-containing protein 1, *CSF-1*: Macrophage colony-stimulating factor 1, *CST5*: Cystatin D, *CX3CL1*: Fractalkine, *CXCL1*: C-X-C motif chemokine 1, *CXCL9*: C-X-C motif chemokine 9, *DNER*: Delta and Notch-like epidermal growth factor-related receptor, *EIF4EBP1*: Eukaryotic translation initiation factor 4E-binding protein 1, *FGF-23*: Fibroblast growth factor 23, *FGF-5*: Fibroblast growth factor 5, *GDNF*: Glial cell line-derived neurotrophic factor, *HGF*: Hepatocyte growth factor, *IL-10*: Interleukin-10, *IL-10RB*: Interleukin-10 receptor subunit beta, *IL-15RA*: Interleukin-15 receptor subunit alpha, *IL-17C*: Interleukin-17C, *IL-6*: Interleukin-6, *LAP TGFß1*: Latency-associated peptide transforming growth factor beta-1, *LIF-R*: Leukemia inhibitory factor receptor, *MCP-1*: Monocyte chemotactic protein 1, *MCP-3*: Monocyte chemotactic protein 3, *NT-3*: Neurotrophin-3, *OPG*: Osteoprotegerin, *PD-L1*: Programmed cell death 1 ligand 1, *SLAMF1*: Signaling lymphocytic activation molecule, *TNFRSF9*: Tumor necrosis factor receptor superfamily member 9, *TNFSF14*: Tumor necrosis factor ligand superfamily member 14, *TRANCE*: TNF-related activation-induced cytokine, *TWEAK*: Tumor necrosis factor (Ligand) superfamily, member 1, *uPA*: Urokinase-type plasminogen activator, *VEGF-A*: Vascular endothelial growth factor A.

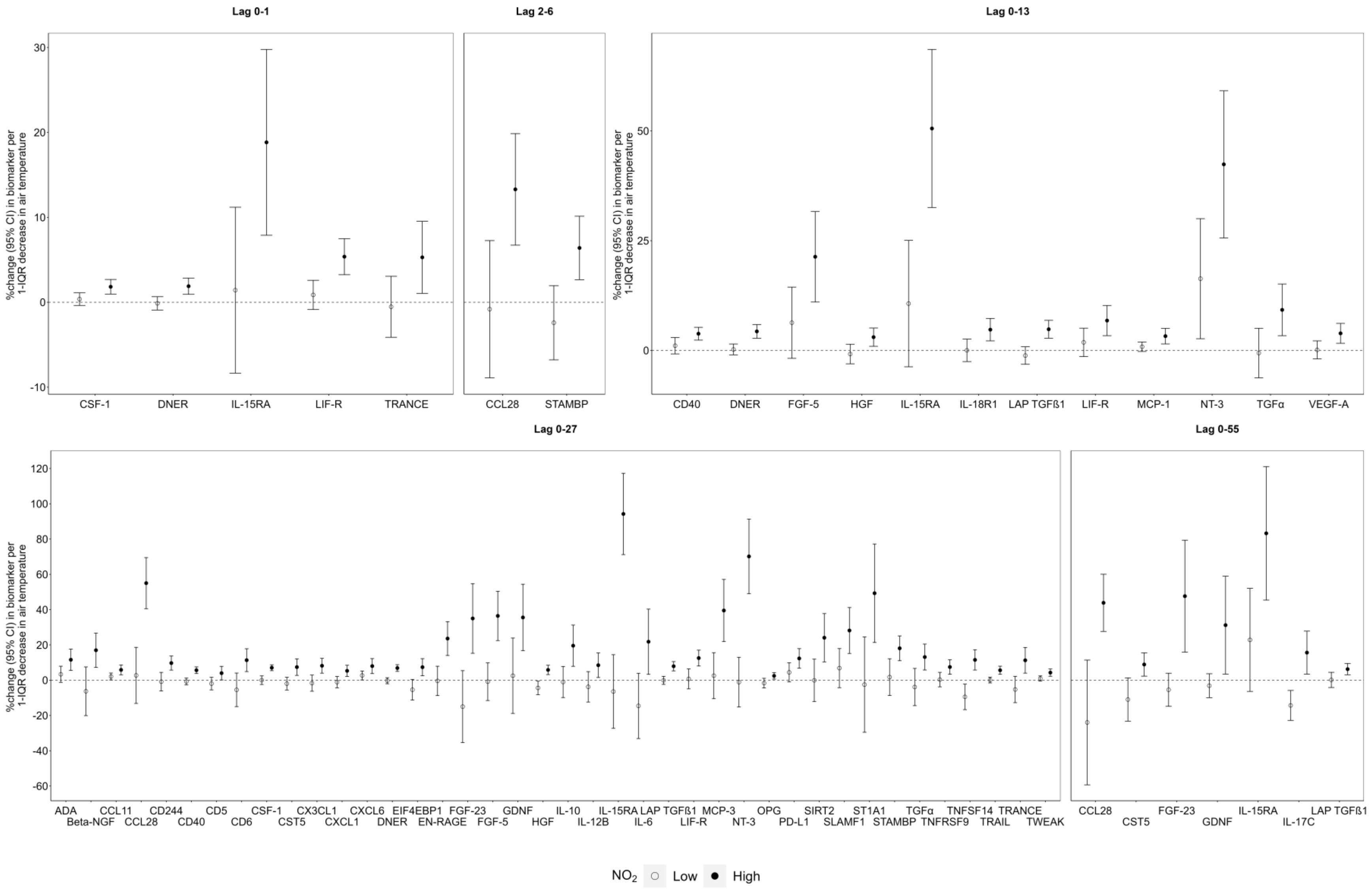

Lag 0-1
Lag 2-6
Lag 0-13
Lag 0-27
Lag 0-55
%change (95% CI) in biomarker per 1-IQR decrease in air temperature
CSF-1
DNER
IL-15RA
LIF-R
TRANCE
CCL28
STAMBP
CD40
FGF-5
HGF
IL-18R1
LAP TGFß1
MCP-1
NT-3
TGFα
VEGF-A
ADA
Beta-NGF
CCL11
CD244
CD5
CD6
CST5
CX3CL1
CXCL1
CXCL6
EIF4EBP1
EN-RAGE
FGF-23
GDNF
IL-10
IL-12B
IL-6
OPG
PD-L1
SIRT2
SLAMF1
ST1A1
TNFRSF9
TNFSF14
TRAIL
TWEAK
IL-17C
NO2 Low High

**Figure S12. Short- and medium-term effects of air temperature on biomarkers of subclinical inflammation per 1-IQR decrease significantly modified by $NO_2$.**

Note: 1-IQR decrease was 9.2°C for lags 0-1 days, 8.9°C for lags 2-6 days, 8.4°C for lags 0-13 days, 9.0°C for lags 0-27 days, and 9.4°C for lags 0-55 days. The Y-axis ranges are not comparable in terms of short- and medium-term effects, because visibility reasons. *ADA*: Adenosine deaminase, *Beta-NGF*: Beta-nerve growth factor, *CCL11*: Eotaxin, *CCL28*: C-C motif chemokine 28, *CD244*: Natural killer cell receptor 2B4, *CD40*: CD40L receptor, *CD5*: T-cell surface glycoprotein CD5, *CD6*: T cell surface glycoprotein CD6 isoform, *CSF-1*: Macrophage colony-stimulating factor 1, *CST5*: Cystatin D, *CX3CL1*: Fractalkine, *CXCL1*: C-X-C motif chemokine 1, *CXCL6*: C-X-C motif chemokine 6, *DNER*: Delta and Notch-like epidermal growth factor-related receptor, *EIF4EBP1*: Eukaryotic translation initiation factor 4E-binding protein 1, *EN-RAGE*: Protein S100-A12, *FGF-23*: Fibroblast growth factor 23, *FGF-5*: Fibroblast growth factor 5, *GDNF*: Glial cell line-derived neurotrophic factor, *HGF*: Hepatocyte growth factor, *IL-10*: Interleukin-10, *IL-12B*: Interleukin-12 subunit beta, *IL-15RA*: Interleukin-15 receptor subunit alpha, *IL-17C*: Interleukin-17C, *IL-18R1*: Interleukin-18 receptor 1, *IL-6*: Interleukin-6, *LAP TGFß1*: Latency-associated peptide transforming growth factor beta-1, *LIF-R*: Leukemia inhibitory factor receptor, *MCP-1*: Monocyte chemotactic protein 1, *MCP-3*: Monocyte chemotactic protein 3, *NT-3*: Neurotrophin-3, *OPG*: Osteoprotegerin, *PD-L1*: Programmed cell death 1 ligand 1, *SIRT2*: SIR2-like protein 2, *SLAMF1*: Signaling lymphocytic activation molecule, *ST1A1*: Sulfotransferase 1A1, *STAMBP*: STAM-binding protein, *TGFα*: Transforming growth factor alpha, *TNFRSF9*: Tumor necrosis factor receptor superfamily member 9, *TNFSF14*: Tumor necrosis factor ligand superfamily member 14, *TRAIL*: TNF-related apoptosis-inducing ligand, *TRANCE*: TNF-related activation-induced cytokine, *TWEAK*: Tumor necrosis factor (Ligand) superfamily, member 1, *VEGF-A*: Vascular endothelial growth factor A.

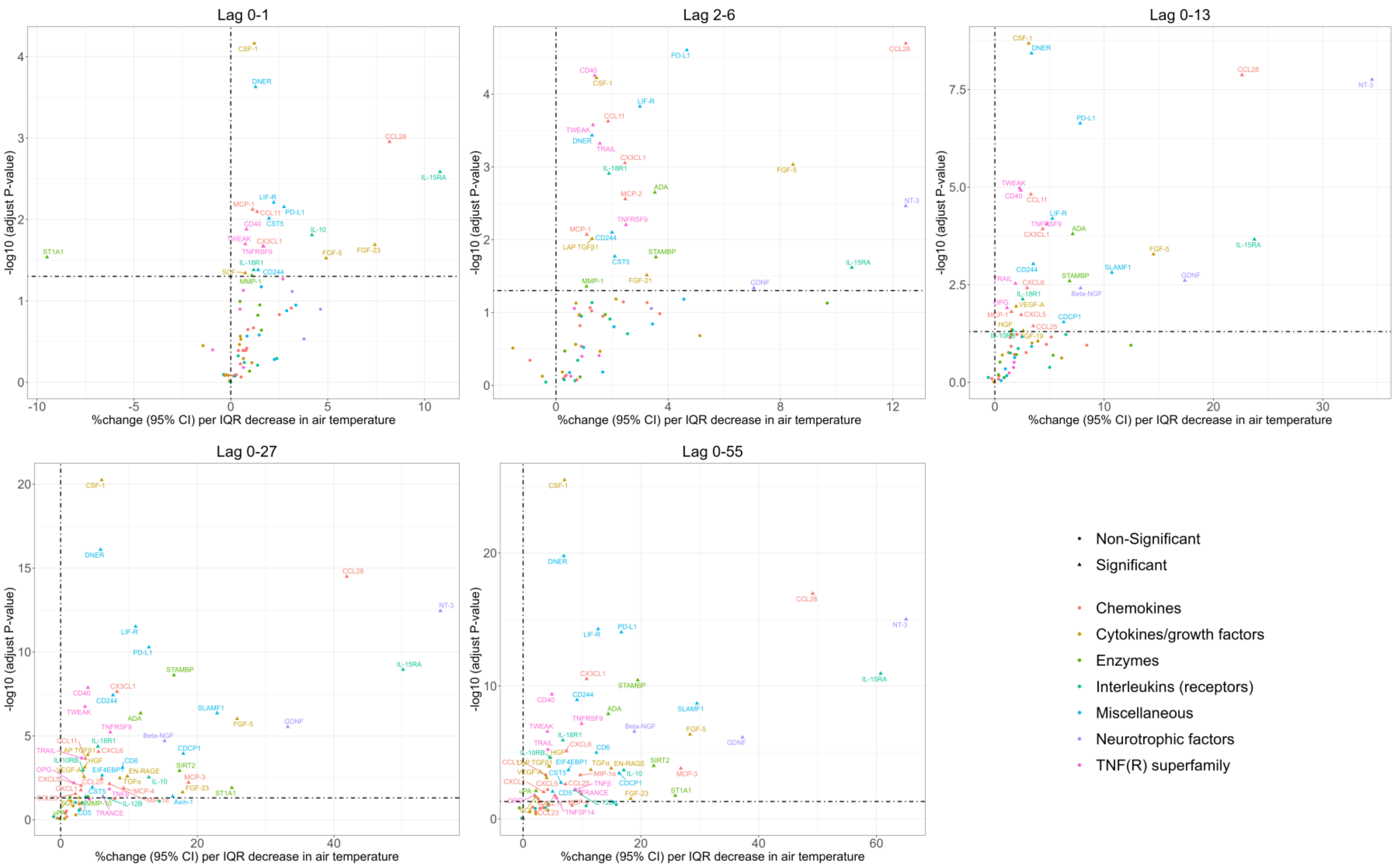
Lag 0-1
Lag 2-6
Lag 0-13
Lag 0-27
Lag 0-55
-log10 (adjust P-value)
%change (95% CI) per IQR decrease in air temperature
Non-Significant
Significant
Chemokines
Cytokines/growth factors
Enzymes
Interleukins (receptors)
Miscellaneous
Neurotrophic factors
TNF(R) superfamily

**Figure S13. Sensitivity analysis (participants with CRP values > 10 mg/L were excluded): Significant associations between short- and medium-term exposures to air temperature per 1-IQR decrease with biomarkers of subclinical inflammation.**

**References**

1. Wolf, K.; Schneider, A.; Breitner, S.; Meisinger, C.; Heier, M.; Cyrys, J.; Kuch, B.; von Scheidt, W.; Peters, A., Associations between short-term exposure to particulate matter and ultrafine particles and myocardial infarction in Augsburg, Germany. *International journal of hygiene and environmental health* **2015,** *218*, (6), 535-42; DOI:10.1016/j.ijheh.2015.05.002.

2. Chen, K.; Breitner, S.; Wolf, K.; Hampel, R.; Meisinger, C.; Heier, M.; von Scheidt, W.; Kuch, B.; Peters, A.; Schneider, A., Temporal variations in the triggering of myocardial infarction by air temperature in Augsburg, Germany, 1987-2014. *European heart journal* **2019,** *40*, (20), 1600-1608; DOI:10.1093/eurheartj/ehz116.

3. Mühlberger, N.; Behrend, C.; Stark, R.; Holle, R. J. I. B. E. M. B., Database-supported identification and entry of drug data in health studies -experiences with the IDOM software. [In German.]. **2003,** *34*, 601-611.
